# Purified CDT toxins and a clean deletion within the CDT locus provide novel insights into the contribution of binary toxin in cellular inflammation and *Clostridioides difficile* infection

**DOI:** 10.1371/journal.ppat.1012568

**Published:** 2024-09-19

**Authors:** Kateryna Nabukhotna, Shannon L. Kordus, John A. Shupe, Rubén Cano Rodríguez, Anna Smith, Julia K. Bohannon, M. Kay Washington, D. Borden Lacy

**Affiliations:** 1 Department of Biochemistry, Vanderbilt University, Nashville, Tennessee, United States of America; 2 Department of Pathology, Microbiology, and Immunology, Vanderbilt University Medical Center, Nashville, Tennessee, United States of America; 3 Department of Anesthesiology, Vanderbilt University Medical Center, Nashville, Tennessee, United States of America; 4 Department of Veterans Affairs, Tennessee Valley Healthcare System, Nashville, Tennessee, United States of America; Texas A&M University, UNITED STATES OF AMERICA

## Abstract

*Clostridioides difficile* is a spore-forming pathogen and the most common cause of healthcare-associated diarrhea and colitis in the United States. Besides producing the main virulence factors, toxin A (TcdA) and toxin B (TcdB), many of the common clinical strains encode the *C*. *difficile* transferase (CDT) binary toxin. The role of CDT in the context of *C*. *difficile* infection (CDI) is poorly understood. Inflammation is a hallmark of CDI and multiple mechanisms of inflammasome activation have been reported for TcdA, TcdB, and the organism. Some studies have suggested that CDT contributes to this inflammation through a TLR2-dependent priming mechanism that leads to the suppression of protective eosinophils. Here, we show that CDT does not prime but instead activates the inflammasome in bone marrow-derived dendritic cells (BMDCs). In bone marrow-derived macrophages (BMDMs), the cell binding and pore-forming component of the toxin, CDTb, alone activates the inflammasome and is dependent on K^+^ efflux. The activation is not observed in the presence of CDTa and is not observed in BMDMs derived from *Nlrp3*^-/-^ mice suggesting the involvement of the NLRP3 inflammasome. However, we did not observe evidence of CDT-dependent inflammasome priming or activation *in vivo*. Mice were infected with R20291 and an isogenic CRISPR/Cas9-generated R20291 Δ*cdtB* strain of *C*. *difficile*. While CDT contributes to increased weight loss and cecal edema at 2 days post infection, the relative levels of inflammasome-associated cytokines, IL-1β and IL-18, in the cecum and distal colon are unchanged. We also saw CDT-dependent weightloss in *Nlrp3*^-/-^ mice, suggesting that the increased weightloss associated with the presence of CDT is not a result of NLRP3-dependent inflammasome activation. This study highlights the importance of studying gene deletions in the context of otherwise fully isogenic strains and the challenge of translating toxin-specific cellular responses into a physiological context, especially when multiple toxins are acting at the same time.

## Author summary

*Clostridioides difficile* is a pathogen causing life-threatening diarrhea. *C*. *difficile* transferase (CDT) toxin is present in many strains associated with severe disease but its role during *C*. *difficile* infection is unclear. In this study, we show that the pore forming subunit, CDTb, activates the inflammasome in myeloid-derived murine cells. While we found that CDT contributes to weight loss in a *C*. *difficile* mouse infection model, this was independent of the inflammasome function. This study highlights the importance of studying gene deletions in the context of otherwise fully isogenic strains and the challenge of translating findings in tissue culture models to animal infections. However, it does reveal an additive role for CDT in the tissue edema that occurs in the murine model of acute infection and is consistent with the idea that CDT contributes to the severity of CDI symptoms.

## Introduction

*Clostridioides difficile* is a Gram-positive, spore-forming, anaerobic bacterium and the leading cause of hospital-associated diarrhea in the United States [1,2]. *C*. *difficile* spores present in the environment survive ingestion via the fecal-oral route and germinate within the small intestine. While the colon is typically resistant to *C*. *difficile* colonization, the dysbiosis associated with the use of antibiotics provides an opportunity for *C*. *difficile* to colonize and grow. Toxigenic strains can promote *Clostridioides difficile* infection (CDI) with clinical symptoms of mild to severe diarrhea and, in some cases, severe sequelae such as pseudomembranous colitis, toxic megacolon, sepsis, and multiple organ dysfunction syndrome [3]. It is estimated that *C*. *difficile* caused approximately 223,900 infections in hospitalized patients and 12,800 deaths in the United States in 2017 alone, and thus, CDI presents a tremendous burden to the US healthcare system [4].

Symptomatic infection is associated with the production of the large clostridial toxins A (TcdA) and B (TcdB) [5,6], and strains expressing only CDT are rarely associated with human disease. However, clinical reports suggest that humans infected with strains expressing CDT along with TcdA and/or TcdB frequently experience more severe clinical outcomes [7]. For example, CDT is found in the NAP1/BI/ribotype 027 *C*. *difficile* strains associated with several *C*. *difficile* epidemics. Consistent with clinical surveillance, studies in mice suggest that CDT does not cause symptoms of disease in the absence of TcdA and TcdB [8,9] but the contribution of CDT in the presence of the other toxins remains unclear [7].

CDT is a binary toxin composed of two protein components: CDTa and CDTb [10]. Once secreted, CDTb binds the lipolysis stimulated lipoprotein receptor (LSR) on the host cell surface and undergoes proteolytic cleavage as well as oligomerization to form a heptameric prepore. One molecule of CDTa, the enzymatic component of CDT, then binds the CDTb heptamer [11]. The CDTa-(CDTb)_7_ complex, hereafter denoted as CDT, is then internalized via endocytosis, and endosomal acidification triggers translocation and delivery of CDTa through the CDTb pore into the host cell. Inside the cell, the ADP-ribosyltransferase (ADPRT) domain of CDTa caps globular actin with an ADP-ribose moiety preventing actin polymerization. Actin depolymerization causes formation of microtubule protrusions that have been shown to increase *C*. *difficile* adherence by ~ 5-fold on the surface of epithelial cells under anaerobic conditions, a mechanism that may enhance *C*. *difficile* virulence [12].

Very little is known about the host immune response to CDT. For TcdA and TcdB, inflammasome activation is one axis of the host innate immune response that contributes to the immunopathology associated with *C*. *difficile* infection [13]. The inflammasome is an innate immune response consisting of a cytosolic multiprotein cascade that regulates host immune homeostasis. Upon inflammasome activation via a sensor molecule (e.g., NLRP3, pyrin) and an adaptor molecule (e.g., ASC), pro-caspase-1 (pro-CASP1) gets processed into caspase-1 (CASP1) which subsequently cleaves precursor pro-inflammatory cytokines (e.g., pro-IL-1β, pro-IL18) into their biologically active forms (e.g., IL-1β, IL18) [14–16]. TcdA and TcdB are known activators of the NLRP3 and pyrin inflammasomes [17–20]. Activation of the inflammasome also requires an initial priming signal during which molecules such as lipopolysaccharide (LPS) upregulate transcription of the inflammasome components (e.g., *Nlrp3*, *Il1b*) via NF-kB-dependent pathways. Previous work exploring the role of discrete Toll-like receptors (TLRs) in NF-kB activation suggested that CDT can act as a priming signal in a TLR2/6-dependent manner [21,22]. Here, we further investigated CDT’s role in host inflammasome enhancement. Our study indicates that CDT does not prime the inflammasome, but instead can activate the inflammasome through the pore-forming activity of CDTb *in vitro*. However, when evaluating the physiological significance of this finding *in vivo*, we did not discover inflammasome-dependent phenotypes induced by CDT.

## Results

### CDT does not prime the inflammasome

**Fig 1:**
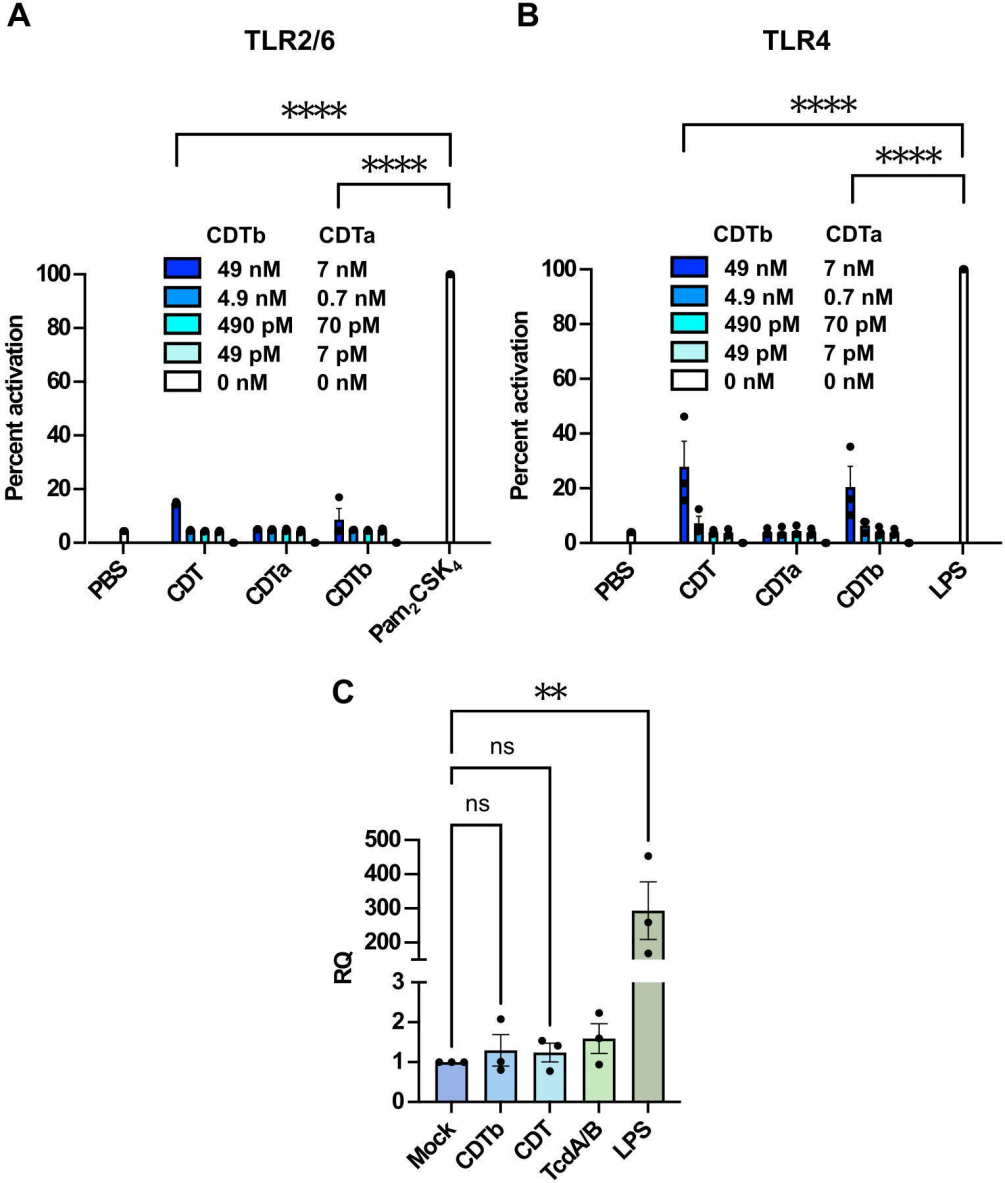
CDT does not prime the inflammasome. (**A**) CDT does not activate TLR2/6 reporter cells. TLR2/6 reporter HEK293 cells were treated with 10 ng/mL of positive control Pam_2_CSK_4_, CDTa, CDTb, or CDT (a mixture of CDTa and CDTb) and incubated for 16 hours at 37°C. NF-κB activation was measured by spectrophotometric detection of secreted alkaline phosphatase (SEAP). (**B**) CDT does not activate TLR4 reporter cells. TLR4 reporter HEK293 cells were treated with 100 ng/mL of positive control LPS and CDTa, CDTb, or CDT following the same methodology as in (**A**). In both (**A**) and (**B**), positive control readings were reported as 100% activation. Treatments are reported as percentages relative to the positive control. Bars show mean ± SEM of three independent biological experiments (*n* = 3). Each independent experiment represents the average of technical duplicates. A two-way ANOVA with Šídák’s multiple comparisons test was used to calculate statistical significance (**** *P* < 0.0001). (**C**) CDT does not prime *Il1b* transcription in primary BMDCs. BMDCs were treated with 700 pM CDTb (monomer), 100 pM CDT, 100 pM CDTa, 7 pM TcdA/B, or 100 ng/mL of LPS for 4 hours at 37°C. Upon RNA extraction and cDNA conversion, qRT-PCR was performed. Differences between *Il1b* and *Gapdh* were measured in each sample. *Il1b* gene expression was normalized to the untreated sample. RQ = transcript fold change. Bars show mean ± SEM of three independent biological experiments (*n* = 3). Each independent experiment represents the average of technical duplicates. A one-way ANOVA with Tukey’s multiple comparisons test was used to calculate statistical significance (** *P* = 0.0019, ns > 0.9999).

CDTa and CDTb were expressed and purified separately as recombinant proteins and mixed to form a toxin that was active in inducing cytopathic responses on epithelial cells (S1 Fig). However, in our experiments using the HEK293 TLR2/6 reporter cell line, neither CDT (the mixture of CDTa and CDTb) nor individual components (CDTa or CDTb by themselves) were able to elicit a TLR2/6 dependent signal (Fig 1A). We also explored whether CDT interacts with TLR4 by using a HEK293 TLR4 reporter cell line, since the absence of the TLR4 pathway somewhat reduced the priming signal in the previous study [21]. While there was some response with the application of 7 nM CDT or 49 nM CDTb alone, it was significantly lower relative to the positive control (Fig 1B). In addition, there was no significant activation of the NF-kB pathway relative to the positive control in more dilute conditions.

We next tested whether CDT induces *Il1b* gene expression in primary mouse bone marrow-derived dendritic cells (BMDCs). Using 100 pM concentrations of CDT, the transcript amount of *Il1b* gene encoding pro-IL-1β assessed by qRT-PCR in CDTb- and CDT-treated cells barely differed from the mock-treated cells (Fig 1C). As expected, TcdA and TcdB combined also did not change transcription levels of *Il1b*. Based on these data, we conclude that CDT does not prime the inflammasome.

### CDTb activates the NLRP3 inflammasome in myeloid-derived murine cells

**Fig 2:**
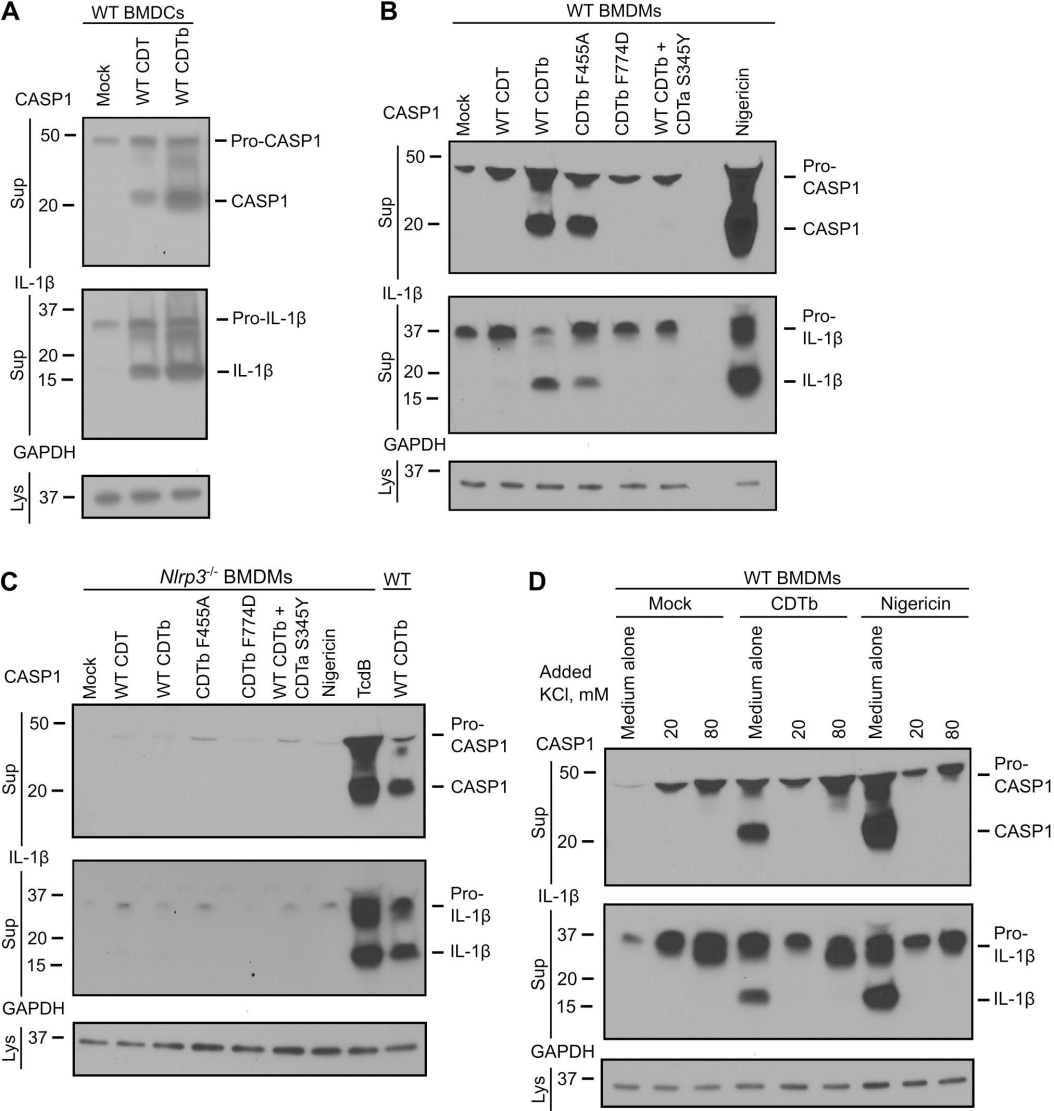
CDTb activates the NLRP3 inflammasome in myeloid-derived murine cells. (**A**) WT BMDCs were primed with 100 ng/mL of LPS (3 h) followed by CDT (4 h) treatments as indicated. Assay was performed with 70 nM CDTb (monomer) and 14 nM CDTa using WT proteins. (**B**) WT and (**C**) *Nlrp3*-deficient BMDMs were treated as indicated using the same methodology as in (**A**). Assay was performed with 70 nM CDTb and 14 nM CDTa using either WT or mutant proteins, and 10 μM of positive control nigericin (45 min). TcdB (**C**, well 8) was added as a positive control for *Nlrp3*-deficient BMDMs (370 pM, 2 h), and a supernatant sample from CDTb-treated WT BMDMs (**C**, well 9) was run on the same gel with samples from *Nlrp3*-deficient BMDMs to demonstrate that when the signal from WT cells appears on the blot, the signal from *Nlrp3*^-/-^ cells is absent. (**D**) WT LPS-primed BMDMs (100 ng/mL, 3 h) were treated with increasing concentrations of extracellular KCl directly before stimulation with 10 μM nigericin (45 min) and 70 nM CDTb (4 h). Secretion of cleaved caspase-1 and mature IL-1β in the precipitated supernatants, and expression of GAPDH in the lysates were assessed by Western blotting. In each panel, representative Western blots from three (*n* = 3) independent experiments are shown.

Given that TcdA and TcdB can trigger inflammasome assembly [17–20], we decided to test whether CDT can also activate the inflammasome. We indeed observed inflammasome activation in LPS-primed BMDCs in response to CDT as assessed by the secretion of cleaved caspase-1 and cleaved IL-1β into the cellular supernatant (Fig 2A). Notably, the effect was still present and seemed to be enhanced when CDTb was added alone. This suggested that inflammasome activation could be a response to CDTb pore formation.

We next tested if the same response could be observed in primary mouse bone marrow-derived macrophages (BMDMs), a cell type classically used to study NLRP3 inflammasome biology [23]. While treatment with CDTb alone resulted in detection of cleaved caspase-1 and IL-1β in cellular supernatants, the presence of CDTa inhibited this signal (Fig 2B). The use of three CDT mutants (single amino acid point mutants in either CDTa or CDTb) (S1 Fig) allowed us to further probe the CDTb-dependency of this phenotype. Inclusion of a CDTa S345Y mutant, known to be deficient in the ADPRT catalytic activity [24], did not restore inflammasome activation, suggesting that the enzymatic activity of WT CDTa is not responsible for the inhibition. Further, there was no pro-caspase-1 or pro-IL-1β cleavage observed after treating the cells with CDTb F774D, a mutation that prevents CDTb cell surface binding [25]. Notably, the CDTb F455A mutant, disrupted in the Phi clamp, a ring of phenylalanines important for cargo translocation [25], was still able to activate the inflammasome. This result suggested that the activation arises due to pore formation, as mutations within the Phi clamp do not impair conductivity of ions across the channel [26]. As many pore forming toxins are known to specifically activate the NLRP3 inflammasome [27], we hypothesized that CDTb activates the inflammasome via the NLRP3/IL-1β axis. Cleavage of pro-caspase-1 and pro-IL-1β was not observed in BMDMs derived from *Nlrp3*^-/-^ mice, consistent with this hypothesis (Fig 2C). Positive controls in this experiment included nigericin (a classical NLRP3 activator), TcdB (which is able to activate pyrin-dependent inflammasomes in BMDMs [18]), and a sample from the CDTb-treated wildtype BMDM cells.

A series of follow-up experiments were performed to further explore and strengthen the above conclusions. To evaluate the concentration dependence, we performed a titration of CDTb on BMDMs and discovered that caspase-1 cleavage remains detectable at lower concentrations, down to ~4.4 nM of activated monomer (the equivalent of 0.63 nM heptamer) (S2A Fig). The activation was not apparent when cells were treated with 0.5 or 1 nM CDTb. Second, we noted that CDTb treatment itself does not provide the priming signal as caspase-1 cleavage was not detected in the absence of LPS priming (S2B Fig, wells 1 and 2). Third, since the experiments in Fig 2 were performed with a 1.4 molar excess of CDTa (10 nM CDTb heptamer with 14 nM CDTa), we tested whether CDTa would have an inhibitory effect when added at an equimolar ratio (10 nM CDTb heptamer with 10 nM CDTa). We saw that the presence of CDTa at either concentration inhibited inflammasome activation in BMDMs (S2B Fig, wells 3, 4, and 5). We also showed that CDTa specifically inhibits CDTb-mediated inflammasome activation, as co-treatment of nigericin and CDTa did not inhibit the cleavage signal (S2B Fig, wells 7 and 8). To test whether inflammasome activation was occurring thru the ATP-P_2_X_7_-NLRP3 pathway, we pretreated BMDMs with A438079, a P_2_X_7_ antagonist. Blocking P_2_X_7_ did not prevent CDTb-dependent caspase-1 processing (S2C Fig). Moreover, inflammasome activation was still observed when CDTb was added to BMDMs primed with Pam_3_CSK_4_, a TLR2/1 ligand (S2D Fig). This excludes the possibility of non-canonical inflammasome activation thru LPS. Finally, we showed that treatments of BMDMs with CDT and CDTb did not impact cell viability at the 4-hour timepoint used for the inflammasome assays as the intracellular ATP levels remained unchanged across multiple CDT concentrations (S2E Fig).

Previous structural studies demonstrated that CDTa interacts with CDTb by being centered on top of the CDTb heptamer [11]. Knowing the importance of cellular K^+^ efflux in NLRP3 inflammasome activation [16], we hypothesized that the transit of ions through the CDTb pore gets blocked in the presence of CDTa. Indeed, when K^+^ efflux was prevented by creating a high extracellular KCl environment in the medium, CDTb was no longer able to activate the inflammasome (Fig 2D).

### CDT contributes to mouse weight loss at day 2 post *C*. *difficile* infection

Our next goal was to evaluate whether the CDTb-dependent NLRP3 inflammasome activation observed *in vitro* is relevant *in vivo*. To do this, we first generated a *cdtB* deletion in the R20291 (ribotype 027) strain using CRISPR/Cas9 mutagenesis. Colony PCR was performed to validate gene deletion, and the genome of the *cdtB* mutant strain was sequenced to confirm that the deletion did not introduce additional mutations within the organism (sequencing reads were deposited to NCBI Sequencing Read Archive under accession number PRJNA1053392). There were no growth differences between R20291 and the mutant strain (S3A Fig). A previous study reported that modifications within *cdtR*, the regulatory gene of CDT production located within the CDT locus (the region encoding for *cdtA* and *cdtB* genes) can influence TcdA and TcdB production [28]. We therefore tested whether the *cdtB* deletion impacted the secretion of TcdA and/or TcdB. We saw that both strains secreted similar amounts of TcdA and TcdB *in vitro* (S3B–S3E Fig). While we did note a decrease in the level of CDTa, this is not expected to impact CDT toxicity in the absence of CDTb.

**Fig 3:**
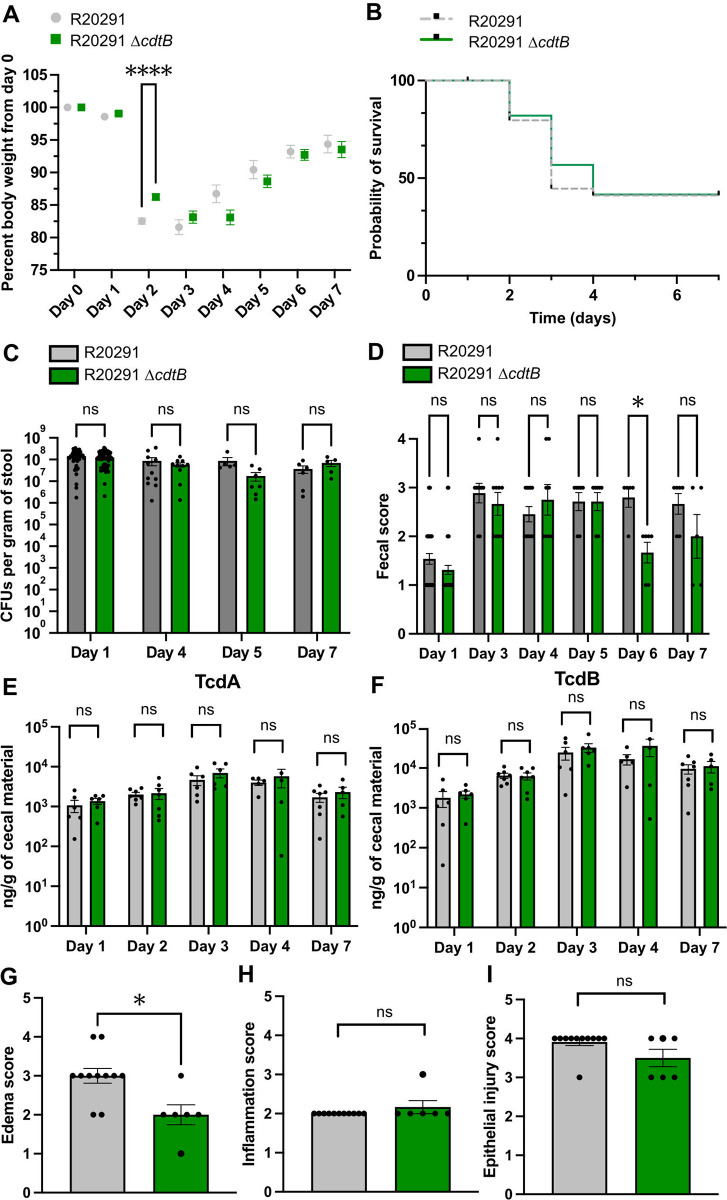
CDT contributes to mouse weight loss at day 2 post *C*. *difficile* infection. (**A**) WT C57BL6J mice were infected by oral gavage on day 0 with the indicated strains and monitored daily for weight loss. Each point represents the mean ± SEM of the group at each day. Number of animals: day 1—*n* = 59 (R20291), *n* = 51 (R20291 Δ*cdtB*); day 2—*n* = 53 (R20291), *n* = 45 (R20291 Δ*cdtB*); day 3—*n* = 21 (R20291), *n* = 26 (R20291 Δ*cdtB)*; day 4—*n* = 12 (R20291), *n* = 15 (R20291 Δ*cdtB*); days 5, 6, & 7—*n* = 7 (R20291), *n* = 7 (R20291 Δ*cdtB*). Mixed-effects analysis with Šídák’s multiple comparisons test was used to calculate statistical significance (**** *P* < 0.0001; ns > 0.3530). (**B**) Survival curve of mice infected with the indicated strains (*P* = 0.7316). Number of animals is the same as in (**A**). Log-rank (Mantel-Cox) comparison test was used with statistical significance set at a *P* value of <0.05. (**C**) Stool samples were collected on indicated days post-infection, plated on selective TCCFA medium, and *C*. *difficile* colony forming units (CFUs) were enumerated. Bars represent mean ± SEM of the group; dots represent an individual mouse within the group. Number of animals: day 1—*n* = 48 (R20291), *n* = 53 (R20291 Δ*cdtB*); day 4—*n* = 11 (R20291), *n* = 10 (R20291 Δ*cdtB*); day 5—*n* = 5 (R20291), *n* = 7 (R20291 Δ*cdtB*); day 7—*n* = 6 (R20291), *n* = 5 (R20291 Δ*cdtB*). Mixed-effects analysis with Šídák’s multiple comparisons test was used to calculate statistical significance (ns > 0.4107). (**D**) Stool samples were collected on indicated days post-infection and scored based on the following criteria: 1 = normal, dry, well-formed stool; 2 = well-formed, moist, discolored stool; 3 = soft, discolored, often mucousy stool; 4 = wet tail, watery diarrhea. Bars represent mean ± SEM of the group; dots represent an individual mouse within the group. Number of animals: day 1—*n* = 39 (R20291), *n* = 45 (R20291 Δ*cdtB*); day 3—*n* = 9 (R20291), *n* = 9 (R20291 Δ*cdtB)*; day 4—*n* = 11 (R20291), *n* = 8 (R20291 Δ*cdtB*); day 5—*n* = 7 (R20291), *n* = 7 (R20291 Δ*cdtB*); day 6—*n* = 5 (R20291), *n* = 6 (R20291 Δ*cdtB)*; day 7—*n* = 6 (R20291), *n* = 5 (R20291 Δ*cdtB*). Mixed-effects analysis with Šídák’s multiple comparisons test was used to calculate statistical significance (* *P* = 0.0217; ns > 0.5337). The weight loss, survival curves, and CFU quantification reflect the data from seven independent studies; fecal scores reflect data from four independent studies. (**E**) TcdA and (**F**) TcdB were quantified in cecal material of euthanized mice during the indicated timepoints of the infection. Bars represent mean ± SEM of the group; dots represent an individual mouse within the group. Number of animals: day 1—*n* = 6 (R20291), *n* = 6 (R20291 Δ*cdtB*); day 2—*n* = 7 (R20291), *n* = 7 (R20291 Δ*cdtB)*; day 3—*n* = 6 (R20291), *n* = 6 (R20291 Δ*cdtB*); day 4—*n* = 5 (R20291), *n* = 5 (R20291 Δ*cdtB*); day 7—*n* = 7 (R20291), *n* = 5 (R20291 Δ*cdtB)*. Two-way ANOVA with Šídák’s multiple comparisons test was used to calculate statistical significance (**E**—ns > 0.5110; **F**—ns > 0.2068). Experiments with cecal toxin concentration measurements were independently performed 2 times (with 3 animals per two groups on each day). Ceca from the infected mice were preserved for histopathological scoring at 2 days post infection for signs of (**G**) edema, (**H**) inflammation, and (**I**) epithelial damage. Bars represent mean ± SEM of the group; dots represent an individual mouse within the group. Number of animals: *n* = 11 (R20291), *n* = 6 (R20291 Δ*cdtB*). Mann-Whitney test with two-tailed *P* value was used to calculate statistical significance (**G** -* *P* = 0.0159; **H**—ns = 0.3529; **I**—ns = 0.0987). Experiments with histopathology analysis at 2 days post infection were independently performed 3 times (with 2–5 animals per 2 groups).

Next, we used the cefoperazone model of mouse *C*. *difficile* infection to test whether the presence of CDTb has a role in pathogenesis. Briefly, wildtype C57BL/6J mice were treated with 0.5 mg/ml cefoperazone in their drinking water for 5 days and then allowed to recover with regular water for 2 days. Mice were then orally gavaged with 10^3^ spores of *C*. *difficile* R20291 or mutant R20291 Δ*cdtB* (lacking CDTb but expressing the rest of the toxins (TcdA^+^TcdB^+^CDTa^+^CDTb^-^). The differences between infected mice were evaluated for up to seven days by comparing mouse weight loss and survival, *C*. *difficile* colonization, and stool scores. Some mice were harvested at 2 days post infection to permit histopathology scoring of the cecal tissue and quantification of neutrophils in the colon. Consistent with previous studies [9,29], mice infected with R20291 lost up to 20% of their body weight by 2 days post infection (Fig 3A). R20291 Δ*cdtB* infection caused significantly less weight loss at 2 days post infection but, by day 3, the R20291 Δ*cdtB*-infected mice lost as much weight as R20291-infected mice. Although not statistically significant, there is a trend indicating that R20291 Δ*cdtB*-infected mice may also have a delayed weight recovery compared to R20291-infected mice 4 days post infection. The absence of CDT did not impact mouse survival (Fig 3B) and bacterial colonization as evidenced by comparable levels of colony forming units (CFUs) of *C*. *difficile* per gram of stool during the infection (Fig 3C). All mice elicited similar signs of diarrhea (Fig 3D) as tracked by daily visual assessment of moisture, color, and consistency of stool summarized in a previously defined scoring system [9].

We quantified protein levels of both TcdA and TcdB in the cecal material of the infected mice during the first 4 days of the infection using a quantitative sandwich ELISA assay [30]. Both strains secreted similar amounts of TcdA and TcdB at every timepoint of the infection (Fig 3E and 3F) indicating that the observed delay in weight loss at day 2 is driven by CDT and not by differences in levels of TcdA/TcdB.

In addition, histopathological phenotypes in hematoxylin & eosin (H&E) stained cecal tissues 2 days post infection were examined by a board-certified gastrointestinal pathologist using previously defined criteria [31]. We saw decreased cecal edema in R20291 Δ*cdtB*- infected mice compared to R20291- infected mice (Figs 3G, S4A and S4B) correlating with the decreased weight loss. However, neither cecal inflammation (Figs 3H, S4A and S4B) nor epithelial injury (Figs 3I, S4A and S4B) were different across the groups. Overall, these data show that CDT contributes to mouse weight loss and cecal edema at day 2 post *C*. *difficile* infection.

### CDT does not impact *C*. *difficile* infection in the inflammasome-dependent manner

**Fig 4:**
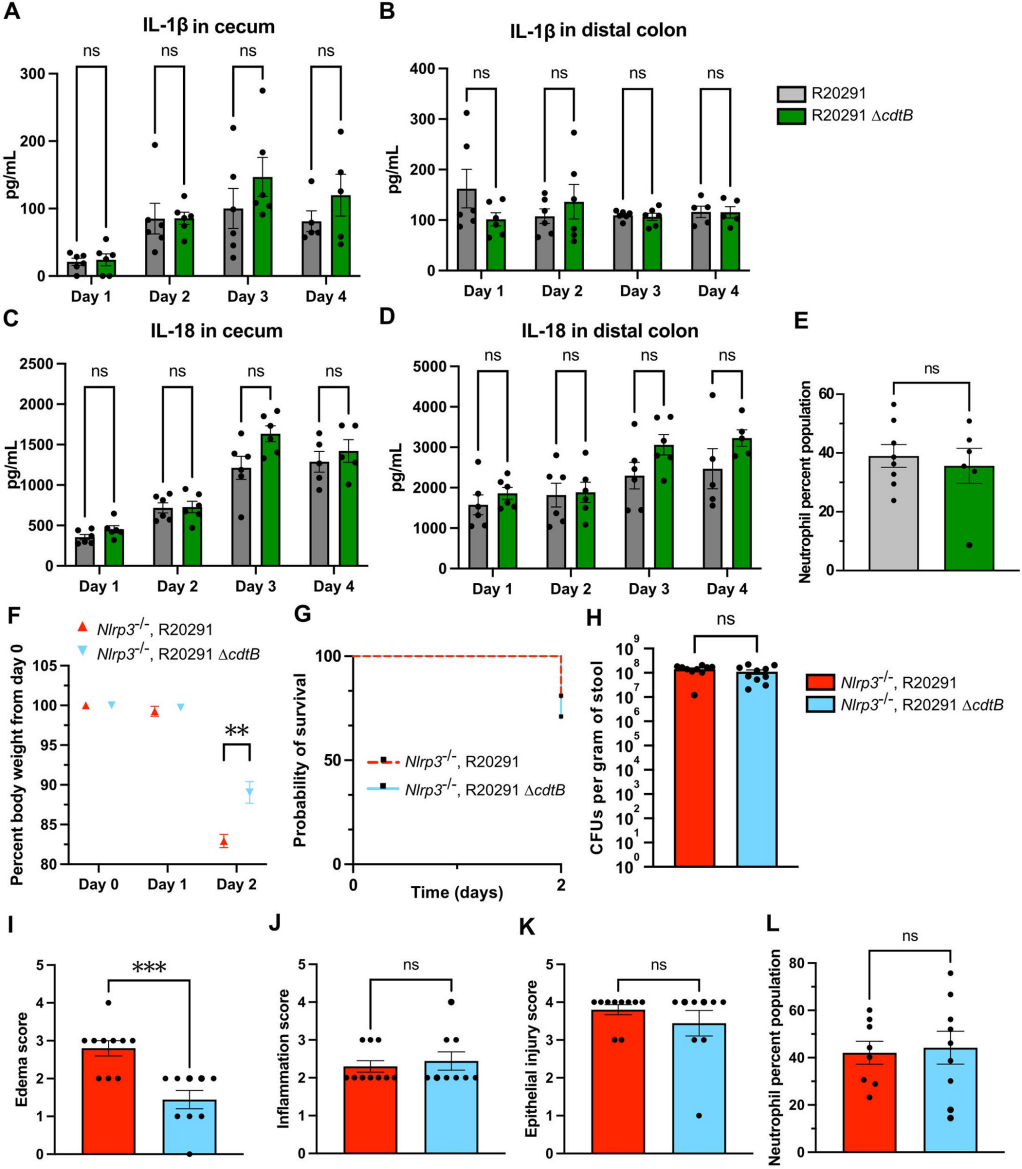
CDT does not impact *C*. *difficile* infection in the inflammasome-dependent manner. IL-1β in 50 μg of (**A**) cecal and (**B**) colonic tissues, and IL-18 in 20 μg of (**C**) cecal and (**D**) colonic tissues were quantified from WT C57BL6J mice infected with the indicated strains during the indicated timepoints of the infection. Bars represent mean ± SEM of the group; dots represent an individual mouse within the group. Number of animals: days 1, 2 & 3—*n* = 6 (R20291), *n* = 6 (R20291 Δ*cdtB*); day 4—*n* = 5 (R20291), *n* = 5 (R20291 Δ*cdtB)*. Mixed-effects analysis with Šídák’s multiple comparisons test was used to calculate statistical significance (**A**—ns > 0.7368; **B**—ns > 0.5513; **C**—ns > 0.1446; **D**—ns > 0.3393). Experiments with tissue cytokine measurements were independently performed 2 times (with 3 animals per two groups on each day). (**E**) Lamina propria cells isolated from the colons of the infected mice at 2 days post infection were labeled with anti-CD11b and anti-Ly-6G antibodies and analyzed by flow cytometry. Percentage of CD11b^+^Ly-6G^+^ neutrophils among live cells as determined by side (SSC) and forward (FSC) scatters are shown. Bars represent mean ± SEM of the group; dots represent an individual mouse within the group. Number of animals: *n* = 8 (R20291), *n* = 6 (R20291 Δ*cdtB*). Mann-Whitney test with two-tailed *P* value was used to calculate statistical significance (ns = 0.9497). Experiments with flow cytometry analysis at 2 days post infection were independently performed 3 times (with 2–5 animals per 2 groups). (**F**) *Nlrp3*^-/-^ C57BL6J mice were infected by oral gavage on day 0 with the indicated strains and monitored daily for weight loss. Each point represents the mean ± SEM of the group at each day. Number of animals: days 1 & 2—*n* = 10 (R20291), *n* = 9 (R20291 Δ*cdtB*). Two-way ANOVA with Šídák’s multiple comparisons test was used to calculate statistical significance (** *P* = 0.0057; ns = 0.9068). (**G**) Survival curve of mice infected with the indicated strains (*P* = 0.6147). Number of animals is the same as in (**F**). Log-rank (Mantel-Cox) comparison test was used with statistical significance set at a *P* value of <0.05. (**H**) Stool samples were collected 1 day post infection, plated on selective TCCFA medium, and *C*. *difficile* colony forming units (CFUs) were enumerated. Bars represent mean ± SEM of the group; dots represent an individual mouse within the group. Number of animals: *n* = 10 (R20291), *n* = 10 (R20291 Δ*cdtB*). Mann-Whitney test with two-tailed *P* value was used to calculate statistical significance (ns = 0.3822). Ceca from the infected mice were preserved for histopathological scoring at 2 days post infection for signs of (**I**) edema, (**J**) inflammation, and (**K**) epithelial damage. Bars represent mean ± SEM of the group; dots represent an individual mouse within the group. Number of animals: *n* = 10 (R20291), *n* = 9 (R20291 Δ*cdtB*). Mann-Whitney test with two-tailed *P* value was used to calculate statistical significance (**G**—*** *P* = 0.0009; **H**—ns = 0.8142; **I**—ns = 0.4985). (**L**) Lamina propria cells isolated from the colons of the infected mice at 2 days post infection were labeled and analyzed by flow cytometry as described in (**E**). Bars represent mean ± SEM of the group; dots represent an individual mouse within the group. Number of animals: *n* = 8 (R20291), *n* = 9 (R20291 Δ*cdtB*). Mann-Whitney test with two-tailed *P* value was used to calculate statistical significance (ns = 0.9089). All experiments in *Nlrp3*^-/-^ C57BL6J mice (**F**–**L**) were independently performed 3 times (with 2–5 animals per 2 groups).

While histopathological scoring of cecal tissues revealed that edema is most likely responsible for the contribution of CDT to weight loss 2 days post infection, we wanted to further look at inflammasome-associated markers *in vivo*. Thus, we quantified inflammasome-associated cytokines, IL-1β and IL-18, in cecal and colonic tissues of the infected mice during the first 4 days of the infection. The levels of both IL-1β (Fig 4A and 4B) and IL-18 (Fig 4C and 4D) were relatively similar between R20291- and R20291 Δ*cdtB-*infected mice, suggesting that CDT does not influence the levels of inflammasome-associated cytokines at these time points.

The inflammasome is a signaling mechanism that alerts the host to the presence of a potential pathogen and promotes the influx of innate immune cells. Since neutrophils are the primary immediate responders to *C*. *difficile* infection [32], we quantified the number of neutrophils in mouse colons 2 days post infection by flow cytometry. The percentage of colonic neutrophils within the immune cell population was expanded to similar extents in both groups (Figs 4E, S5A and S5B). This observation was supported by comparable levels of calprotectin in both cecal and colonic tissues of infected mice during the first 4 days of the infection (S6 Fig). We also looked at other immune cells in blood by performing complete blood counts during the first 4 days of the infection. Differential analysis showed that the percentages of neutrophils, lymphocytes, and monocytes among all white blood cells were not significantly different between the groups (S7A–S7C Fig). However, we did note that CDT leads to a reduction of type 2 immune cell types eosinophils and basophils at day 1 post infection (S7D–S7E Fig).

Finally, we decided to perform R20291 and R20291 Δ*cdtB* infections in C57BL/6J *Nlrp3*^-/-^ mice. When comparing 2-day long infections in the *Nlrp3* inflammasome knockout animals, we found that the R20291 Δ*cdtB*-infected group still lost less weight than those infected with the wildtype strain (Fig 4F). There was no difference in survival (Fig 4G) and bacterial burden (Fig 4H) between the groups. Differences in histopathological phenotypes in cecal tissues due to CDT were identical to the ones in the wildtype mice. While cecal edema was reduced in R20291 Δ*cdtB*- infected mice compared to R20291- infected mice (Figs 4I, S4C and S4D), cecal inflammation (Figs 4J, S4C and S4D) and epithelial injury (Figs 4K, S4C and S4D) were not. The levels of colonic neutrophils at 2 day post infection did not differ between the groups (Figs 4L, S5C and S5D). Overall, this demonstrates that CDT contributes to cecal edema and weight loss 2 days post R20291 infection through a mechanism that is independent of the NLRP3 inflammasome.

## Discussion

*C*. *difficile* remains one of the urgent threats in the U.S. healthcare system [4], highlighting a tremendous need for characterization of CDI, particularly from the toxin perspective. While the toxins TcdA and TcdB are clearly associated with the symptoms of disease, the role of the CDT binary toxin, when present, remains unclear. Further, the mechanisms of how CDT may be contributing to the severity of infection are also not well understood in a physiologic context. One set of studies has shown that the catalytic function of CDTa is important in promoting the formation of microtubule protrusions which, in turn, may enhance *C*. *difficile* adhesion to the epithelium [12]. Other studies have noted CDTb-mediated roles in inflammasome priming, cell death, and MAIT cell activation [21,33,34]. Here, we provide evidence that CDT does not prime but instead activates the NLRP3 inflammasome *in vitro*.

It is unclear why CDTa completely blocked inflammasome activation in macrophages but not in dendritic cells. The observation could reflect a difference in the kinetics of toxin assembly and entry into cells. For example, if CDTb is assembled and internalized quickly in dendritic cells, some pores could be formed prior to CDTa binding. Alternatively, the observation could reflect differences in the longevity of CDTb pores in the membrane following CDTa delivery. If CDTb pores are more rapidly cleared in macrophages following cargo delivery, this could limit the ion flow needed for NLRP3 inflammasome activation.

In addition to the CDTb-dependent inflammasome activation observed in our *in vitro* data, other studies have reported CDTb-dependent phenotypes in cell culture. Marquardt et. al demonstrated that CDTb induces human mucosal-associated invariant T (MAIT) cell activation in a partially IL-18-dependent manner [34]. The MAIT cell activation required accessory immune cells, suggesting that CDT may target monocytes to release IL-18 into the media. Given the phenotype we observed in murine BMDMs, it is tempting to speculate that CDTb might also activate the NLRP3 inflammasome in human monocytes to facilitate MAIT cell activation. Recent studies from the Barth group reported that CDTb alone is capable of impairing the epithelial integrity of CaCo-2 monolayers [33]. In addition, toxin pore blockers inhibited intoxication of Vero, HCT116, and CaCo-2 cells by CDTb [35]. Overall, these studies support the idea that the binding and pore-forming abilities of CDTb are sufficient to trigger molecular events in a variety of cell types.

Previous work has shown that the presence of TcdA and/or TcdB is required for symptoms in the murine model of CDI; binary toxin by itself in R20291 Δ*tcdA*Δ*tcdB* (TcdA^-^TcdB^-^CDTa^+^CDTb^-^) is not able to cause any symptoms during acute infection [8,9]. Therefore, to test whether CDT-dependent inflammasome activation occurs *in vivo*, we created an R20291 Δ*cdtB C*. *difficile* strain lacking *cdtB*. Given the importance of TcdA/TcdB levels during infection, we took great care to create a clean deletion without polar effects, to ensure that the new strain was otherwise completely isogenic with the parent strain, and to test whether the TcdA and or TcdB levels were altered in either *in vitro* or *in vivo* growth conditions. All assays suggested that the only difference between the two strains was the presence or absence of *cdtB*. The data from comparing two strains in the mouse infection model suggest that CDT contributes to weight loss on the second day of the infection in the mouse infection model and that the contribution is independent of the NLRP3 inflammasome response. We speculate that this phenotype depends on the enzyme function of CDTa and its role in inactivating the actin cytoskeleton. Future studies are needed to test this hypothesis and the possible effect of CDTa activity on epithelial barrier function.

There are several explanations as to why our *in vitro* and *in vivo* data contradict each other. First, in the above *in vivo* studies, we used a R20291 *ΔcdtB* mutant that presumably lacks active binary toxin (considering that CDTa cannot enter host cells without CDTb being present). Our *in vitro* data indicate that CDTa can dampen the inflammasome activation to different extents in certain cell types (BMDCs vs BMDMs). Thus, it is possible that the presence of CDTa in the wild-type strain prevents CDTb-dependent inflammasome activation *in vivo*. Second, the physiological concentration of CDTb and CDTa are not known during *C*. *difficile* infection. While we can see CDTb-induced inflammasome activation *in vitro* at ~ 4.4 nM, it is possible that the concentration of CDTb heptamers does not get this high *in vivo*. Finally, we have considered the possibility that CDT-dependent inflammasome activity is relevant in conditions different from what was tested in this study. In addition to the acute infection setting, the CDT-dependency could be explored at later timepoints, particularly during stages of recovery and recurrence. Questions of whether the pyrin and/or NLRP3-dependent mechanisms of TcdA/TcdB inflammasome activation amplify or compete with that of CDT also merit further study.

The overall role of the NLRP3 inflammasome during CDI is also still unclear. Hasegawa et al. showed that the IL-1β/NLRP3/ASC-axis is responsible for making ASC^-/-^ mice highly susceptible to *C*. *difficile* infection, as an increased number of commensal bacteria were translocated from the gut in the absence of ASC [36]. This implies that NLRP3 signaling is protective during the infection. However, among the mice that survived, there was no difference in weight loss and pathology scores after *C*. *difficile* infection. In contrast, Liu et al reported that the ATP-P_2_X_7_ axis of inflammasome activation is also protective during *C*. *difficile* infection but not in a NLRP3- and pyrin-dependent manner [37]. We note that the Hasegawa and Liu studies were done with the VPI10463 strain of *C*. *difficile* which lacks the gene for CDT.

The Petri laboratory has reported a detrimental role for CDT in the mouse and hamster models of CDI [21,22,38]. In these studies, the authors report that CDT acts as a TLR2/6-dependent priming signal that contributes to inflammasome activation by TcdA/TcdB and that this results in CDT-dependent suppression of protective eosinophils in the colon. The principal difference between our study and this prior work lies in the results of the biochemical cell culture experiments as we did not see evidence of CDT-dependent priming. Here, we note that the protein toxins were expressed and purified in our laboratory using methods identical to those used in prior cryo-electron microscopy reports [11,25]. We added a dedicated step to remove residual endotoxin from the purified CDTa and CDTb proteins to ensure that the immune cells were not responding to a contaminant. We also ensured that the CDTa-(CDTb)_7_ combination was active in a cytopathic rounding assay (S1 Fig). Consistent with the previous study, we saw that mice infected with an R20291 mutant lacking *cdtB*, lost less weight than animals infected with wildtype R20291 [21]. However, in the Cowardin study, mice infected with the mutant strains also had higher survival rates relative to those infected with the parent strain during a 3-day infection. In the follow up study, infection with a strain that had undergone the simultaneous deletion of *cdtA* and *cdtB* in the R20291 background prevented any signs of mouse weight loss or significant disease compared to the R20291 infection [38]. While the result supports a role for CDT in contributing to the severity of CDI, the absence of symptoms is hard to reconcile with the established roles of TcdA and TcdB in causing weight loss in mouse models of infection. It is therefore important to verify that the concentrations of TcdA and TcdB produced under *in vivo* conditions remain unchanged when comparing the phenotypes associated with different mutant strains. In fact, two studies have highlighted that insertion or deletion mutations within the *cdtR* gene that regulates *cdtA* and *cdtB* transcription can result in reduced TcdA and TcdB production and attenuated *C*. *difficile* virulence in mice [28,39]. These data demonstrate that mutagenesis within the CDT locus needs to be carefully performed to avoid disruption of the *cdtR* signaling networks that affect *tcdA* and *tcdB* gene expression. The fact that the concentrations of TcdA and TcdB were unchanged between the two strains over the seven day time course of the experiment (Fig 3E and 3F) support our conclusion that the differences we observed between the two strains were a result of the CDTb deletion.

Despite the differences between the studies, there are some important parallels that merit further exploration. The work by Petri and colleagues suggests that the combination of CDT and TLR2 signaling contributes to suppression of what could be a protective eosinophil response. The paper by Liu and colleagues implicates TLR2 and the ATP-P_2_X_7_ signaling axis in the macrophage response to the organism. We observed a reduction of eosinophils in blood 1 day post infection in R20291-infected mice compared to R20291 Δ*cdtB* -infected mice in our data (S7D Fig), which is supportive of the proposed effect of CDTb on eosinophils. Further studies to evaluate mechanisms of inflammation in the context of all three toxins and the organism are needed to fully understand the complex balance of the protective and detrimental inflammatory responses that affect the successful or unsuccessful response of the host faced with CDI.

## Methods

### Ethics statement

This study was approved by the Institutional Animal Care and Use Committee at Vanderbilt University Medical Center (VUMC). Our laboratory animal facility is AAALAC-accredited and adheres to guidelines described in the Guide for the Care and Use of Laboratory Animals. All animal manipulations were performed in a laminar flow hood. The health of the mice was monitored daily, and severely moribund animals were humanely euthanized.

### Recombinant protein expression and purification

Plasmids for the recombinant expression of CDTb (pBL870, residues 43–876 in a pET28a vector) and CDTa (pBL926, residues 60–463 in a pLM302 vector) were previously described [25]. Both CDTb and CDTa plasmids were transformed into BL21-RIL *E*. *coli* and grown in LB medium supplemented with kanamycin at 37°C at 220 rpm. Cultures were induced at OD_600_ = 0.4–0.6 with 250 μM IPTG per liter of culture. Incubation was further continued at 18°C at 220 rpm overnight (~16–18 hours). Cells were harvested by centrifugation at 10°C for 15 minutes at 3500 rpm and stored at -70°C for further use.

For CDTb purification, the bacterial pellet was resuspended in 20 mM HEPES, pH 8.0, 500 mM NaCl supplemented with DNase I and protease inhibitors (Pepstatin A, Leupeptin, Lysozyme, and PMSF) and lysed by passing through the Avestin EmulsiFlex high-pressure homogenizer. The lysate was clarified by centrifuging at 4°C for 30 minutes at 18,000 rpm and applied to chelating Sepharose resin charged with nickel sulfate. After the lysate was incubated with the resin at 4°C for 1 hour, the resin was collected by gravity flow and washed twice to remove contaminants (first wash with 20 mM HEPES pH 8.0, 100 mM NaCl, 2mM Imidazole; second wash with 20 mM HEPES pH 8.0, 100 mM NaCl, 40 mM Imidazole, pH 8.0). The protein was eluted with 20 mM HEPES pH 8.0, 100 mM NaCl, 150 mM Imidazole, pH 8.0. The eluted fraction was concentrated three-fold using Amicon centrifugal filters and applied to a Q Sepharose column run with a 0–600 mM NaCl gradient (buffer A = 20 mM HEPES, pH 8.0, buffer B = 20 mM HEPES, pH 8.0, 1M NaCl). Fractions of interest were pooled together, concentrated to 1 mL and applied to an S200 column in 20 mM HEPES pH 8.0, 100 mM NaCl buffer. To mimic proteolytic cleavage, CDTb was concentrated and trypsin-activated by incubation with bovine trypsin at 37°C for 45 minutes at a 1:5 (trypsin:toxin) ratio (w/w). PMSF was added at a 1 mM final concentration to quench the reaction, and the sample was applied to an S200 column in 20 mM HEPES pH 8.0, 100 mM NaCl buffer to separate activated CDTb from the cleaved peptide fragment, trypsin and PMSF. Final fractions of 75 kDa CDTb were sterile-filtered, flash frozen in liquid nitrogen, and used in downstream cellular assays. After every purification step, protein fractions were run on an SDS-PAGE gel to assess protein purity.

For CDTa purification, the bacterial pellet was lysed similarly to CDTb, except lysis buffer was supplemented with 10% glycerol. The clarified lysate was immediately applied to chelating Sepharose resin charged with cobalt hexafluoride and allowed to flow through by gravity. The resin was washed with 20 mM HEPES pH 8.0, 100 mM NaCl, 2mM Imidazole, pH 8.0, 10% glycerol. Then the protein was eluted with 20 mM HEPES pH 8.0, 100 mM NaCl, 40 mM Imidazole pH 8.0, 10% glycerol. The eluted fraction was concentrated down to ~3 mL and dialyzed into 20 mM Tris pH 7.0, 100 mM NaCl, 10% glycerol in the presence of PreScission Protease at 4°C overnight. The sample was recovered, diluted to ~10 mL with 50 mM Tris pH 7.0, 100 mM NaCl, 10% glycerol, and run on a SP Sepharose column with a 0–400 mM NaCl gradient (buffer A = 20 mM Tris, pH 7.0, 10% glycerol; buffer B = 20 mM Tris, pH 7.0, 1M NaCl, 10% glycerol). Fractions of interest were pooled together, concentrated to 1 mL and applied to an S200 column equilibrated with 20 mM HEPES pH 8.0, 100 mM NaCl, 10% glycerol. Fractions of interest were pooled together, sterile-filtered, and flash frozen in liquid nitrogen. After every purification step, protein fractions were run on an SDS-PAGE gel to assess protein purity.

CDT point mutants (pBL919, pBL921, pBL1072) were generated as described in [25]. TcdA and TcdB were expressed and purified as described previously [40].

### HEK-Blue TLR activation assay

HEK-Blue hTLR2-TLR6 (hkb-htlr26) and hTLR4 (hkb-htlr4) cells were purchased from InvivoGen and used according to manufacturer instructions. Briefly, cells were grown in DMEM, 4.5 g/L glucose, 2 mM L-glutamine, 10% (v/v) heat-inactivated fetal bovine serum (FBS, Corning 35-011-CV), Pen-Strep (100 U/ml-100 μg/ml), 100 μg/ ml Normocin (and HEK-Blue selection supplement after first two passages). On the day of the assay, TLR2/6 cells were rinsed with pre-warmed PBS, detached with 0.08% trypsin-EDTA (diluted with PBS) and centrifuged at room temperature for 5 minutes at 300 g. Cells were resuspended in pre-warmed PBS and seeded at 50,000 cells per well in a 96-well plate in HEK-Blue Detection medium. TLR4 cells were rinsed with pre-warmed PBS, detached with 3 mL of pre-warmed PBS by pipetting cells up and down, and directly counted without centrifugation. Cells were seeded at 25,000 cells per well in a 96-well plate in HEK-Blue Detection medium. CDT proteins were diluted in PBS, added to the cells, and the plates were incubated at 37°C in 5% CO_2_ for 16 hours. The absorbance at 635 nm was recorded at that time point using BioTek Cytation 5 plate reader. The concentration of positive controls and toxin subunits used are indicated in Fig 1. For these assays, both purified CDTa and CDTb underwent endotoxin removal prep (Thermo 88273) to prevent any potential LPS contamination. The following positive controls were used: Pam_2_CSK_4_ (Invivogen tlrl-pm2s-1), and LPS (Sigma L3024). OD_635_ readings were normalized to the mock condition (Detection medium only). Readings per each condition were averaged and reported relative to the positive control as a percentage (averaged positive control value was treated as 100% activation).

### Animals and housing

Wildtype (JAX stock #664) and *Nlrp3*^-/-^ (JAX stock #21302, [41]) C57BL/6J female mice were purchased between 8–10 weeks of age from Jackson Laboratories. Mice were housed in a pathogen-free room with 12-hour cycles of light and dark with clean bedding and free access to food and water. Cages were changed every two weeks.

### Extraction, differentiation, and plating of bone marrow-derived dendritic cells and macrophages

Femurs were isolated from humanely euthanized mice by CO_2_ inhalation followed by cervical dislocation. Any residual muscles and tissues were removed to expose the bone. The ends of the bone were cut with sterile scissors, and the bone was flushed from both sides with cold complete RPMI medium (supplemented with 10% heat-inactivated FBS, L-glutamine, 100 U/ml-100 μg/ml Pen-Strep, and 50 μM BME). Cells were strained through a 70 μm strainer and spun down at 300 x g for 5 minutes. Upon ACK lysing buffer addition to the cells at RT for 5 minutes, complete RPMI was added, and the cells were spun again at 300 x g for 5 minutes. Cells were seeded at the final concentration of 2x10^5^ cells/mL in 10 mL of complete RPMI and grown at 37°C with 5% CO_2_. For BMDMs, medium contained 20 ng/mL of M-CSF (Peprotech, 315–02); for BMDCs, medium contained 20 ng/mL of GM-CSF (Peprotech, 315–03). On days 3 and 5, half of the medium was removed, and the same volume of fresh complete RPMI with M-CSF or GM-CSF (20 ng/mL final concentration) was added to the cells.

On day 6, BMDMs were rinsed twice with PBS and incubated at 37°C with 5% CO_2_ in the presence of 5 mL of TrypLE dissociation reagent (Thermo Fisher 12604013). Cells were lifted off plates with sterile scrapers, 5 mL of complete RPMI media was added, and the cells were spun down at 310 x g for 5 minutes. After counting, cells were plated at 1x10^6^ cells/well in a 6-well plate in complete RPMI medium and allowed to attach overnight before performing inflammasome activation assay.

BMDCs were plated for the assays on day 7. Medium containing non-adherent cells was transferred into a conical, loosely adherent cells were detached from plates by scraping and added to the same conical. After spinning down and counting, cells were plated at 2.7x10^5^ cells/well in a 48-well plate in complete RPMI medium for measuring *Il1b* transcript for priming assay, and at 2x10^6^ cells/well in a 6-well plate in OPTI-MEM medium for inflammasome activation assay.

### *Il1b* gene expression analysis

BMDCs were treated with the indicated concentration of toxins and LPS (Fig 1) and incubated for 4 hours at 37°C with 5% CO_2._ RNA was isolated using RNeasy Plus Mini Kit (Qiagen 74134) and QIAShredder homogenizers (Qiagen 79656). 75 ng of RNA from each sample was synthesized to cDNA using the QuantiTect Reverse Transcription Kit (Qiagen 205311). Expression of *Il1b* gene was measured using TaqMan-based quantitative Real Time-Polymerase Chain Reaction (qRT-PCR). TaqMan Fast Advanced Master Mix (Thermo Fisher 4444557) and predesigned TaqMan Gene Expression Assays (Thermo Fisher mouse Il1b Mm00434228_m1 and mouse Gapdh Mm99999915_g1, both FAM-MGB) were used. Expression of *Il1b* gene (cycle threshold, Ct) was measured relative to expression of *Gapdh* (ΔCt) in each sample. ΔCt values were compared to ΔCt values in a control (mock treated) sample (ΔΔCt). Data are reported as transcript RQ (relative fold change) defined as 2^−ΔΔ*Ct*^. Data acquisition was performed on QuantStudio 6 Flex Real-Time PCR System.

### Inflammasome activation assay and sample preparation

BMDMs and BMDCs were primed with 100 ng/mL of LPS (Sigma L3024) or 500 ng/mL of Pam_3_CSK_4_ (Invivogen tlrl-pms) in 1 mL of OPTI-MEM medium per well in a 6-well dish and incubated at 37°C with 5% CO_2_ for 3 hours. Toxins were then added to the cells (at concentrations indicated in figure legends) and further incubated at 37°C with 5% CO_2_ for 4 more hours. For the inhibition experiment, the cells were pre-treated with A438079 (500 μM; Tocris Bioscience 2972) for 30 minutes and further intoxicated with CDTb in the presence of the inhibitor. Supernatant was harvested and cells were lysed in RIPA buffer (150 mM NaCl, 1% NP-40, 0.5% Sodium Deoxycholate, 0.1% SDS, 50 mM Tris pH 7.4) supplemented with protease inhibitor tablet (Sigma S8820) and PMSF. Lysates were rotated at 4°C for 15 minutes, spun down and added to 4X SDS buffer with 10% BME in a 3:1 ratio. Supernatant underwent trichloroacetic acid (TCA) precipitation. Briefly, 100 μL of 0.15% deoxycholate was added to 1 mL of supernatant following a 10-minute incubation at room temperature. Then, 50 μL of ice-cold TCA (Sigma T0699) was added to the samples. Samples were precipitated on ice for 30 min and centrifuged at 10,000 x g at 4°C for 15 minutes. The protein pellet was washed one time with 500 μL of ice-cold acetone to remove left-over TCA. After centrifugation, acetone was aspirated and 35 μL of 2X SDS buffer with 10% BME was immediately added to the sample. If sample color changed to green-yellow, 3 μL of Tris pH 8.0 was added to bring the pH up. The samples were stored at -20°C until Western blot analysis was performed.

### Western blot analysis

Samples were boiled at 100°C for 5 minutes and loaded into 4–20% precast polyacrylamide gels (BioRad MiniProtean TGX stain-free gels, 4568094). Proteins were transferred from the gels to polyvinylidene fluoride membrane (Millipore Sigma IPFL00010) at 100 V for 60 minutes. For chemiluminescent detection, the membranes were washed in Tris buffered saline pH 7.6 supplemented with 0.1% Tween 20 (TBST); for fluorescent detection, the membranes were washed in Tris buffered saline pH 7.6 (TBS) without Tween 20. The membranes were blocked for 60 minutes at RT in 3% milk in TBST for chemiluminescent detection and in 3% milk in TBS for fluorescent detection. After blocking, the membranes were incubated at 4°C overnight in 3% milk-TBST containing the following primary antibodies: mouse anti-Caspase-1 at 1:1000 dilution (Adipogen AG-20B-0042-C100), mouse IL-1β at 1:800 dilution (R&D Systems AF-401-NA), mouse GAPDH at 1:5000 dilution (Cell Signaling 2118S), anti-TcdA antibody at a 1:1000 dilution (clone PCG4.1, Novus Biologicals NB600-1066), anti-TcdB antibody at a 1:1000 dilution (Exalpha ACdTB), anti-CDTa antibody at a 1:1000 dilution (Exalpha ACdBTSA), and anti-CDTb antibody at a 1:1000 dilution (Exalpha ACdBTSB). The next day, the membranes were washed three times in TBST for 10 minutes. After washing, the membranes were incubated at RT for 60 minutes in 3% milk-TBST containing the following secondary antibodies at a 1:10000 dilution: goat anti-rabbit HRP conjugated IgG (Jackson Immunoresearch 111-035-144), goat anti-mouse HRP conjugated IgG (Jackson Immunoresearch 115-035-062), donkey anti-goat HRP conjugated IgG (Jackson Immunoresearch 705-035-003), anti-mouse IgG DyLight 680 conjugate (Cell Signaling 5470S), and goat anti-chicken IgY DyLight 680 conjugate (Invitrogen SA5-10074). The membranes were washed three times in TBST for 10 minutes. For fluorescent detection, membranes were washed one more time in TBS for 10 minutes and imaged immediately on BioRad ChemiDoc MP Imaging System. For chemiluminescent detection, the membranes were incubated in HRP substrate for 5 minutes (Millipore Sigma WBKLS0500).

### Luminescent cell viability assay

CellTiter-Glo assay was performed according to manufacturer’s instructions (Promega G7570). Briefly, after counting on day 6, BMDMs were plated at 1x10^5^ cells/well in a 96-well plate in complete RPMI medium and allowed to attach overnight. Next day, the cells were treated with the indicated concentrations of CDT proteins or left untreated and incubated for 4 hours at 37°C with 5% CO_2_. CellTiter-Glo Reagent was then added to the equal volume of cell culture medium present in each well. The contents were mixed for 2 minutes on an orbital shaker to induce cell lysis and allowed to incubate at room temperature for 10 minutes to stabilize luminescent signal. Luminescence was recorded on BioTek Cytation 5 plate reader. Relative luminescence units of treatments were normalized to the untreated control (set at 100%) and reported as percentages relative to that.

### Vero-GFP cell rounding assay

To produce Vero-GFP cells, Vero cells (ATCC CCL-81) were transduced with Lentiviral GFP plasmid. Briefly, lentiviral preparations were produced in a 293T (ATCC CRL-3216) cell line using the 2^nd^ generation psPAX2 and pMD2.G packaging system developed by the Trono lab (gifts from Didier Trono, Addgene plasmids #12260 and #12259, respectively). pLenti-GFP-puro was a gift from Eric Campeau and Paul Kaufman (Addgene plasmid #17448 [42]). Lentivirus production was performed per Addgene protocol with one exception (FuGENE 6 (Promega E269A) transfection reagent was used instead of PEI). Vero cells at ~50% confluency were transduced using supernatant from above using Sigma polybrene (TR1003G) protocol at 10 μg/ml final. Cells were incubated overnight, and medium containing viral supernatant was carefully removed and replaced with 10 mL of DMEM with 10% heat inactivated FBS. Cells were allowed to recover for 48 hours, and medium was replaced with DMEM + 10% FBS containing puromycin (10 μg/ml final). Cells surviving antibiotic selection were visualized under fluorescent microscopy for positive GFP expression.

Vero-GFP cells were maintained in DMEM + 10% heat-inactivated FBS and puromycin (10 μg/mL final) and cultured at 37°C with 5% CO_2_. Cells were seeded into 96-well plates at 24,000 cells per well and allowed to grow overnight. Next day, medium was replaced with DMEM + 10% FBS without antibiotic, CDT proteins were added to the cells, and GFP images were taken every 30 minutes on BioTek Cytation 5 plate reader. From these images, the total number of rounded and non-rounded cells were counted as described in [43].

### *C*. *difficile* growth conditions, medium, and strains

*C*. *difficile* strains were grown in a strict anaerobic environment within a COY anaerobic chamber (5% H_2_, 5% N_2_, and 90% CO_2_) statically at 37°C. Strains were grown in BHIS (brain heart infusion-supplemented with 5 g/L yeast extract) medium or TY (30 g/L tryptone and 20 g/L yeast extract) medium, supplemented with 0.1% L-cysteine. For toxin secretion assays, L-cysteine was omitted from the medium. *C*. *difficile* growth medium was supplemented with the following when needed: 0.1% taurocholate [TA], cefoxitin (8 μg/mL), thiamphenicol [Thi] (10 μg/mL), kanamycin [Kan] (50 μg/mL), D-cycloserine (250 μg/mL), lincomycin (20 μg/mL), or 1% D-xylose. *E*. *coli* and *B*. *subtilis* strains were maintained on Lysogeny Broth (LB) at 37°C supplemented with either chloramphenicol [Cam] (34 μg/mL for *E*. *coli* and 5 μg/mL for *B*. *subtilis*), ampicillin [Amp] (100 μg/mL), or tetracycline [Tet] (2.5 μg/mL). For enumerating *C*. *difficile* titers *in vivo*, taurocholate-cefoxitin-cycloserine-fructose agar (TCCFA) contained 0.1% TA, 250 μg/ml D-cycloserine, and 16 μg/ml cefoxitin [44]. All bacterial strains can be found in Table 1. Plasmids and primers used in this study can be found in Tables 2 and 3, respectively.

**Table 1:** Strains used in the study.

Number	Strain name	Relevant features	Source
	***E*. *coli***		
	DH5α	F- Φ80*lacZ*ΔM15 Δ(*lacZYA-argF*) U169 *recA1 endA1 hsdR17* (rK^–^, mK^+^) *phoA supE44* λ–*thi-1 gyrA96 relA1*	Lab stock
	HB101/pRK24	F- *mcrB mrr hsdS20*(rB^–^mB^–^) *recA13 leuB6 ara-13 proA2 lavYI galK2 xyl-6 mtl-1 rpsL20* carrying pRK24	Aimee Shen
	HB101 pJB07 *ΔcdtB*	pJB07 *ΔcdtB* in HB101 pRK24	This work
	MG1655	F- *lambda*- *ilvG*- *rfb-50 rph-1*	ATCC
	MG1655 pJB06	pJB06 (Cam^r^), xylose inducible CRISPR-CAS9, in MG1655	This work
	***Bacillus subtilis***		
	JH BS2	Transconjugant from the mating 630xCU2189 (CU2189::Tn*5397*) Tet^r^	[45]
	JH BS2 pJB06	pJB06 (Cam^r^), in JH BS2	This work
	***C*. *difficile***		
DBLCD6	R20291	Wildtype BI/NAPI/027 from Nottingham Clostridia Research Group	[46]
DBLCD158	R20291 pJB06	DBLCD6 containing pJB06 (Thi^r^), xylose inducible CRISPR-CAS9.	This work
DBLCD166	R20291 *ΔcdtB*	DBLCD6 containing an in-frame deletion of *cdtB*	This work

**Table 2:** Plasmids used in the study.

Number	Plasmid Name	Relevant features	Source
pBL1256	pJB06	Xylose inducible Cas9 expression plasmid for 2-plasmid mutagenesis. (Cam^r^)	[47]
pBL1257	pJB07	Homology arms targeting *pyrE* and gRNA for targeting plasmid for 2-plasmid mutagenesis. (Erm^r^)	[47]
pBL1287	pJB07 Δ*cdtB*	pJB07 homology arms replaced with homology regions for creating in frame *cdtB* deletion and gRNA targeting *cdtB*,	This work

**Table 3:** Primers used in study.

Name	Primer^1^
dCDTb Up For	*TTATCAGGAAACAGCTATGACC*GCGGCCGCGCGTGGAGGATATACTGCAATTAA
dCDTb RFP Rev	*TACTTTCAGTTTAGCGGTCTGGGCGCC*ATTACTTGTACTAGTTTGAGCATATACAGG
dCDTb RFP For	*GGCGCCCAGACCGCTAAACTGAAAGT*AAGGAGTTATTTTACAGGTGGAGAAAATATT
dCDTb down rev.2	*GCCAAGTTGCATGTCTGCAGG*CTCGAGCTCAA AGTGAACTTTTGTTCATGAGAC
CDTb guide 2	*GCATTCAAGGAGGG*GGTACCGATTATTTGGTACCAGAACAGTTTTAGAGCTAGAAATAGC
246 TcdB col for	CTGAGAATGGAGAAATGCAAATAGG
248 TcdB col rev	GCTACTTTTCTGATTCCTCCATCTATTC
315 RFP landing primer	ACTTTCAGTTTAGCGGTCTG
316 cdtB seq rev	GTATGGGAGCAGAAAAAGCC
317 cdtB seq for	GGGATTCTTGGGGTAAAGCA
344 pJB07 guide rev	*AATGCAGGCTTCTTATTTTTAT*GCTAGCACGCGTCTAGTCAGACATCATGCTGATCT
357 pJB07 guide seq.4	ACAGACTTATCCAGGGTG
91 M13R	CAGGAAACAGCTATGAC

^1^All primers are written in 5’-3’ direction. *Italicized* base pairs represent Gibson assembly homologous regions. Underline represents restriction enzyme cut site.

### Plasmid construction of *cdtB* deletion

The *cdtB* deletion strain was created using the two-plasmid mutagenesis system, described elsewhere [47]. gDNA from *C*. *difficile* strain R20291 (DBLCD6) was extracted using the MasterPure Gram Positive DNA Purification Kit (Lucigen). Roughly 700 bp of the upstream homology arm and downstream homology arm of *cdtB* was amplified from R20291 gDNA. dCDTb Up For and dCDTb RFP Rev were used to amplify upstream homology arms and dCDTb RFP For and dCDTb down rev.2 were used to amplify the downstream homology arms. These fragments were inserted into pBL1257 (a gift from Joseph Sorg; Addgene plasmid #190481), previously digested with *Not*I and *Xho*I using Gibson assembly [48]. gRNA was constructed by amplifying pBL1257 using primers CDTb guide 2 and #344 and inserted into pBL1257 containing *cdtB* homology arms digested with *Mlu*I and *Kpn*I. The resulting plasmid was whole plasmid sequenced using Plasmidsaurus to ensure no off-target mutations. pBL1287 was transformed into *E*. *coli* pRK24.

### Plasmid conjugations into R20291

pBL1256 (a gift from Joseph Sorg; Addgene plasmid #190480) was conjugated into *C*. *difficile* strain R20291 using *B*. *subtilis* strain JH BS2. Briefly, pBL1256 isolated from *E*. *coli* DH5α was used to transform *E*. *coli* MG1655 and plasmid purified. The resulting plasmid was transformed into JH BS2. An isolated colony of JH BS2 was inoculated in 2.5 mL LB supplemented with Cam and Tet and grown with aeration for 6 hrs. Simultaneously, a well isolated colony of R20291 was grown overnight, back diluted 1:20 in TY, and grown for 6 hrs. Samples were spread (100 μl) onto TY plates and grown for 24 hrs. The resulting growth was scraped and resuspended in PBS before plating on TY supplemented with Kan and Thi. Resulting colonies were screened for Tet sensitivity. The resulting Thi^r^ Tet^s^ R20291 pBL1256 (DBLCD158) strain was used to create the *cdtB* deletion.

Overnight cultures (~ 16 hrs) of *E*. *coli* pRK24 pBL1287 grown in LB supplemented with Erm and Amp and R20291 pBL1256 grown in TY supplemented with Thi were back diluted 1:20 in the respective media and grown for 6 hrs. *E*. *coli* pRK24 pBL1287 was pelleted at 2,896 x g for 1 min, supernatant removed, and the pellet was transferred to the anaerobic chamber. R20291 pBL1256 was used to resuspend the *E*. *coli* pellets (200 μl) and ~20 μl drops were spotted onto 2 TY supplemented with Thi plates. After 24 hr or 48 hr of growth, the plates were scraped and resuspended in 1 mL TY or PBS and plated onto TY supplemented with Thi, Kan, cefoxitin, and linomycin. Resulting transconjugates were screened for TcdB using primer pair 246 and 248 and for pBL1287 using primer pair 91 and 357.

### Induction of CRISPR Cas9 and whole genome sequencing

Strains containing pBL1287 were struck onto TY supplemented with 1% xylose, lincomycin and Thi. Colonies were screened for deleted *cdtB* using primer pairs 316 and 317 and 315 and 317. The plasmids were cured from strains containing the *cdtB* deletion and the gDNA was extracted using MasterPure Gram Positive DNA Purification Kit (Lucigen). The resulting DNA was sequenced using SeqCoast Genomics. Samples were prepared for whole genome sequencing using an Illumina DNA Prep tagmentation kit and unique dual indexes. Sequencing was performed on the Illumina NextSeq2000 platform using a 300 cycle flow cell kit to produce 2x150bp paired reads. 1–2% PhiX control was spiked into the run to support optimal base calling. Read demultiplexing, read trimming, and run analytics were performed using DRAGEN v3.10.12, an on-board analysis software on the NextSeq2000. Sequencing reads were deposited to NCBI Sequencing Read Archive accession number PRJNA1053392.

### *C*. *difficile* growth curve generation

*C*. *difficile* strains were plated on BHIS supplemented with TA plates from glycerol stocks. Next day, 5 mL TY were inoculated and grown statically for 18 hours. Overnight cultures were subcultured to an OD_600_ of 0.01 in TY medium and 200 μL of culture were added per well in a pre-reduced 96-well plate (Corning 3788). The plate was inserted into Stratus kinetic microplate reader (Cerillo), and readings were taken every hour for 15 hours. Each condition was done in technical triplicate. Readings were subtracted from the TY medium only.

### C. difficile toxin secretion in vitro

*C*. *difficile* strains were plated on BHIS supplemented with TA plates from glycerol stocks. Next day, 5 mL TY were inoculated and grown statically for 16 hours. Overnight cultures were seeded to an OD_600_ of 0.01 in 5 mL of TY medium and allowed to grow for ~22 hours. Bacterial cultures were centrifuged at 3220 x g for 5 minutes, supernatants were filtered through a 0.2 μm cellulose acetate membrane (VWR 5141273) and added to 4X SDS buffer with 10% BME in a 3:1 ratio. Samples were normalized based on the terminal OD_600_ reading. Samples were subjected to Western blot analysis as described above.

### *C*. *difficile* spore preparation

*C*. *difficile* strains were plated on BHIS supplemented with TA plates from glycerol stocks. Next day, 5 mL TY were inoculated and grown statically for 16 hours. 2 mL of overnight culture were inoculated into 40 mL of Clospore medium [49] which was then grown for 5 days anaerobically. On day 6, the suspension was centrifuged at 1980 x g for 20 minutes at 10°C, and the pellet was washed three times in cold sterile water. Spores were resuspended in 1 mL of sterile water and heat treated at 65°C for 20 minutes to eliminate vegetative cells. Spores were quantified by performing serial dilutions on BHIS supplemented with TA plates. Spore stocks were stored at 4°C until use.

### Mouse model of *C*. *difficile* infection

Prior to antibiotic treatment, mice were assimilated to the new facility for one week to reduce stress. Mice were given 0.5 mg/ml cefoperazone (Sigma C4292) in sterile drinking water for 5 days [31]. Antibiotic water was refreshed every other day to prevent antibiotic breakdown. After 5 days, mice were switched to regular water to recover for 2 days before inoculation. Mice were orally gavaged with 10^3^ *C*. *difficile* spores. Mouse weight and symptoms were recorded daily, and stool samples were also collected daily, except for the second day post infection due to severe diarrhea in mice and thus lack of fecal pellets for extended amount of time. To assess severity of diarrhea, the following criteria were used to visually score the appearance of fecal pellets: 1 = normal, dry, well-formed stool; 2 = well-formed, moist, discolored stool; 3 = soft, discolored, often mucousy stool; 4 = wet tail, watery diarrhea. To enumerate *C*. *difficile* titers *in vivo*, stool was then weighed, macerated in 1 mL sterile PBS pH 7.4, and plated anaerobically (in serial dilutions) on TCCFA plates for 19 hours at 37°C. At the end of the studies, mice were humanely euthanized by CO_2_ inhalation. If an animal was found moribund or its weight loss was more than 20% of the initial mass, the weightloss and tissue samples were included but the animal was humanely euthanized and marked as dead. If an animal was found dead, the weights and tissues were not included. For histopathology scoring of the cecal tissue of mice harvested at 2 days post infection, the cecum was excised from each animal, laid flat on sterile Whatman filter paper, and fixed in 10% neutral buffered formalin for 24 hours at 4°C. After fixation, tissues were transferred to 70% ethanol and cut into pieces using the CecAx preservation method [50]. Tissues were submitted in 70% ethanol to the Translational Pathology Shared Resource at Vanderbilt University Medical Center for paraffin embedding and serial sectioning. Cecum sections were stained with hematoxylin & eosin using a staining kit (Abcam ab245880) and submitted to a board-certified gastrointestinal pathologist to score edema, inflammation, and epithelial damage from 0–4 [31]. In brief, the following methods were used: the edema scores: 0—no edema; 1—mild edema with minimal multifocal submucosal expansion; 2—moderate edema with moderate multifocal sub-mucosal expansion; 3—severe edema with severe multifocal sub-mucosal expansion; 4—same as score 3 with diffuse sub-mucosal expansion. Inflammation scores: 0—no inflammation; 1—minimal multifocal neutrophilic inflammation; 2—moderate multifocal neutrophilic inflammation (greater submucosal involvement); 3—severe multifocal to coalescing neutrophilic inflammation (greater submucosal ± mural involvement; 4—same as score 3 with abscesses or extensive mural involvement. Epithelial damage scores: 0—no epithelial changes; 1—minimal multifocal superficial epithelial damage (vacuolation, apoptotic figures, villus tip attenuation/necrosis); 2—moderate multifocal superficial epithelial damage (vacuolation, apoptotic figures, villus tip attenuation/necrosis); 3—severe multifocal epithelial damage (same as above) +/− pseudomembrane (intraluminal neutrophils, sloughed epithelium in a fibrinous matrix); 4—same as score 3 with significant pseudomembrane or epithelial ulceration (focal complete loss of epithelium).

Experiments with histopathology and flow cytometry analyses at 2 days post infection were independently performed 3 times (with 2–5 animals per 4 groups). Experiments with a terminal endpoint at 7 days post infection (with sacrifice days between 2 and 4 days based on moribundity) were independently performed 2 times (with 10 animals per two groups). Experiments with tissue cytokine, and cecal toxin concentration measurements with daily terminal endpoints between 1 and 4 days post infection were independently performed 2 times (with 3 animals per two groups on each day).

### Flow cytometry analysis

For quantification of neutrophils in the colonic tissue of mice harvested at 2 days post infection, the colon was excised from each animal, flushed with PBS, and opened longitudinally. Colon was washed twice in HBSS, cut into small pieces (~0.7 cm), and incubated in buffered HBSS supplemented with 5 mM EDTA and 1mM DTT (Thermo Fisher R0861) at 37°C for 15 minutes. Supernatant was poured off and tissues were further incubated in buffered HBSS supplemented with 5 mM EDTA at 37°C for 10 minutes. After HBSS washes, tissues were further minced with curved scissors about 50 times and incubated in HBSS supplemented with 20% FBS, 0.5 mg/mL collagenase D (Millipore Sigma 11088858001) and 10 mg/mL DNAse I (Sigma DN25) at 37°C for 30 minutes. Supernatant containing lamina propria cells was strained through a 70 μm cell strainer and centrifuged for 20 minutes at 10°C at 650 x g. Cell pellet was resuspended in PBS supplemented with 2 mM EDTA and 2% FBS, counted using BioRad TC20 Automated Cell Counter and resuspended in PBS to ~ 10^6^ cells/200 μL. Cells were incubated with CD16/CD32 Fc block (BD Pharmingen 553142), and 100 μL of cell suspension were stained with 0.1 mg/mL of FITC rat anti-mouse Ly-6G (BD Pharmingen 551460) and PE rat anti-mouse CD11b (Invitrogen 12-0112-83) at RT for 15 minutes. Isotype-matched antibodies were used for control staining. Cells were washed with PBS, centrifuged for 10 minutes at 4°C at 300 x g, and resuspended in 100 μL of PBS. Data were collected on Accuri C6 Plus Flow Cytometer and analyzed with Accuri C6 software. Side and forward scatters were used to gate on live cells, and neutrophils were reported as a double positive CD11b^+^Ly-6G^+^ percentage of the gated cells.

### Quantification of TcdA and TcdB in cecal material

Levels of TcdA and TcdB were measured using a quantitative sandwich ELISA assay developed elsewhere [30]. Briefly, cecal material was harvested from euthanized mice and snap frozen. The contents were resuspended in 1.5 mL of PBS, macerated, and incubated on ice for 30 minutes. For TcdA, the samples were normalized to 10 mg/mL of cecal material, and for TcdB to 0.5 mg/mL. The slurries were then serially diluted 2-fold in PBS-T + 2% BSA and added to ELISA plates. For TcdA, A1D8 capture and A1C3 detection nanobodies were used. For TcdB, B0D10 capture and B0E2 detection nanobodies were used. To account for plate-to-plate variability, rTcdA or rTcdB standard curves were included on each plate.

### Quantification of IL-1β, IL-18, and calprotectin

Levels of IL-1β, IL-18, and calprotectin in cecal and colonic tissues were analyzed via ELISA. Small pieces of cecum and distal colon were rinsed with PBS, snap frozen and weighted. Tissues were homogenized in T-PER reagent (Fisher 78510) at a ratio of ~10 mg of tissue to 200 μL of the reagent supplemented with protease inhibitor tablet (Sigma S8820) and PMSF. The homogenates were centrifuged for 5 minutes at 4°C at 10,000 x g. The supernatant was collected, and total protein concentration in the supernatant was measured using BCA assay (Thermo 23225). DuoSet ELISA kits were used to measure IL-1β (R&D Systems DY401) in 50 μg of tissue lysate, IL-18 (R&D Systems DY7625) in 20 μg of tissue lysate, and calprotectin in 20 μg of tissue lysate (R&D Systems DY8596) according to the manufacturer’s instructions.

### Complete blood count analysis

Blood was collected via cardiac puncture into tubes supplemented with EDTA (Greiner 450532) right after mouse euthanasia. Whole blood was immediately submitted to the Translational Pathology Shared Resource at Vanderbilt University Medical Center for complete blood counts. Differential analysis shows neutrophils, lymphocytes, monocytes, eosinophils, and basophils as a percentage within the total number of white blood cells.

### Statistical analysis

All statistical analyses and graphical representations were performed using GraphPad Prism software (GraphPad software Corporation, Inc, CA, USA). For statistical comparison of two groups with one independent variable, unpaired t-test or Mann-Whitney test with two-tailed *P* value were used to calculate statistical significance (statistical significance set at a *P* value of <0.05). For statistical comparison of more than two groups with one independent variable, a one-way ANOVA with Tukey’s multiple-comparisons test was used (statistical significance set at a *P* value of <0.05). For statistical comparison of two groups with two independent variables, two-way ANOVA or mixed-effects analysis with Šídák’s multiple comparisons tests were used (threshold for *P* value comparison set to 0.05). The Log-rank (Mantel-Cox) multiple comparison test was used for survival curve comparisons (statistical significance set at a *P* value of <0.05).

## Supporting information

S1 Fig Functional validation of WT and mutant CDT. Point mutations in the Phi clamp (CDTb F455A), cell binding domain (CDTb F774D) or CDTa enzyme domain (CDTa S345Y) render WT CDT inactive in a Vero-GFP cell rounding assay. Assay was performed with 7 nM CDTb and 1 nM CDTa using either WT or mutant proteins. Each data point represents mean ± SEM of three independent biological experiments at a specific timepoint (*n* = 3).
(TIF)

S2 Fig Control experiments in BMDMs. (**A**) WT BMDMs were primed with 100 ng/mL of LPS (3 h) followed by CDTb (4 h) treatments as indicated. (**B**) Non-primed (wells 1 and 2) or 3-hour primed with 100 ng/mL of LPS (wells 3, 4, 5, 7, and 8) WT BMDMs were further treated for 4 hours as indicated. Assay was performed with 70 nM CDTb:10 nM CDTa (7:1), 70 nM CDTb:14 nM CDTa (5:1) or 10 μM nigericin (45 min). (**C**) WT BMDMs were primed with 100 ng/mL of LPS (3 h). The cells were then pretreated with 500 μM of A438079 for 30 min followed by addition of CDTb (4 h). (**D**) WT BMDMs were primed with 500 ng/mL of Pam_3_CSK_4_ (3 h) followed by CDT (4 h) treatments as indicated. Assay was performed with 70 nM CDTb and 14 nM CDTa. Secretion of cleaved caspase-1 in the precipitated supernatants, and expression of GAPDH in the lysates were assessed by Western blotting. A representative Western blot from two (*n* = 2) independent experiments is shown. (**E**) WT BMDMs were treated with indicated CDT and CDTb concentrations for 4 hours. ATP was measured using a CellTiter-Glo Luminescence Assay.
(TIF)

S3 Fig *In vitro* characterization of R20291 and R20291 Δ*cdtB*. (**A**) *In vitro* growth curves of each strain subcultured to OD_600_ 0.01 in TY medium. The OD_600_ was measured at every hour for 15 hours. Each data point represents mean ± SEM of three biological experiments (*n* = 3). (**B**) CDTa, (**C**) CDTb, (**D**) TcdA, and (**E**) TcdB were quantified in culture supernatants. Briefly, each strain was subcultured to OD_600_ 0.01 in TY medium and grown for 22 hours. Samples were normalized based on the terminal OD_600_ reading. Representative Western blots of three (*n* = 3) independent experiments are shown. Densitometry measurements were performed in BioRad Image Lab software. Average band intensity of the mutant strain was reported in reference to average band intensity of WT strain treated as “1”. For these assays, fluorescent secondary antibodies were used to ensure reliable band intensity quantification. Bars represent mean ± SEM of the group. Unpaired t-test was used to calculate statistical significance (**B**—** *P* = 0.0038; **D**—ns = 0.4560; **E**—ns = 0.4959).
(TIF)

S4 Fig Representative H&E stained tissues used for histopathological scoring. Representative images of H&E stained cecal tissues from (**A**) R20291-, (**B**) R20291 Δ*cdtB-*, (**C**) *Nlrp3*^-/-^, R20291-, and (**D**) *Nlrp3*^-/-^, R20291 Δ*cdtB*- infected mice. Red arrows point to areas of edema, yellow arrows point to inflammation, and green arrows point to epithelial injury. Scale bar = 50 μm.
(TIF)

S5 Fig Representative flow cytometry plots. Representative flow cytometry plots of stained cells isolated from colons of (**A**) R20291-, (**B**) R20291 Δ*cdtB-*, (**C**) *Nlrp3*^-/-^, R20291-, and (**D**) *Nlrp3*^-/-^, R20291 Δ*cdtB*- infected mice. The plots are gated on live (as determined by FSC vs. SSC) CD11b^+^Ly-6G^+^ cells from the colonic lamina propria.
(TIF)

S6 Fig Calprotectin in (A) cecal and (B) colonic tissues was quantified from WT C57BL6J mice infected with the indicated strains during the indicated timepoints of the infection. Bars represent mean ± SEM of the group; dots represent an individual mouse within the group. Number of animals: days 1, 2 & 3—n = 6 (R20291), n = 6 (R20291 ΔcdtB); day 4—n = 5 (R20291), n = 5 (R20291 ΔcdtB). Mixed-effects analysis with Šídák’s multiple comparisons test was used to calculate statistical significance (A—ns > 0.6726; B—ns > 0.8039). Experiments with tissue calprotectin measurements were independently performed 2 times (with 3 animals per two groups on each day).
(TIF)

S7 Fig White blood cell differentials following complete blood counts. Differential analysis of white blood cells—(**A**) neutrophils, (**B**) lymphocytes, (**C**) monocytes, (**D**) eosinophils, and (**E**) basophils–in WT C57BL6J mice infected with the indicated strains during the indicated timepoints of the infection. Cell populations are shown as percentages of all white blood cells. Bars represent mean ± SEM of the group; dots represent an individual mouse within the group. Number of animals: day 1—*n* = 6 (R20291), *n* = 5 (R20291 Δ*cdtB)*; day 2—*n* = 11 (R20291), *n* = 8 (R20291 Δ*cdtB*); day 3—*n* = 5 (R20291), *n* = 6 (R20291 Δ*cdtB)*; day 4—*n* = 3 (R20291), *n* = 4 (R20291 Δ*cdtB*). Mixed-effects analysis with Šídák’s multiple comparisons test was used to calculate statistical significance (**A**—ns > 0.2959; **B**—ns > 0.2335; **C**—ns > 0.8704; **D**—** *P* = 0.0061, ns > 0.4512; **E**—** *P* = 0.0031, * *P* = 0.0138, ns > 0.9976). Experiments with complete blood counts were independently performed 3 times (with 2–5 animals per 2 groups).
(TIF)
