# Supplementary figures and images for: Purified CDT toxins and a clean deletion within the CDT locus provide novel insights into the contribution of binary toxin in cellular inflammation and Clostridioides difficile infection
Source: PLoS Pathog. 2024 Sep 19;20(9):e1012568. doi: 10.1371/journal.ppat.1012568 (PMC11444381; doi:10.1371/journal.ppat.1012568)

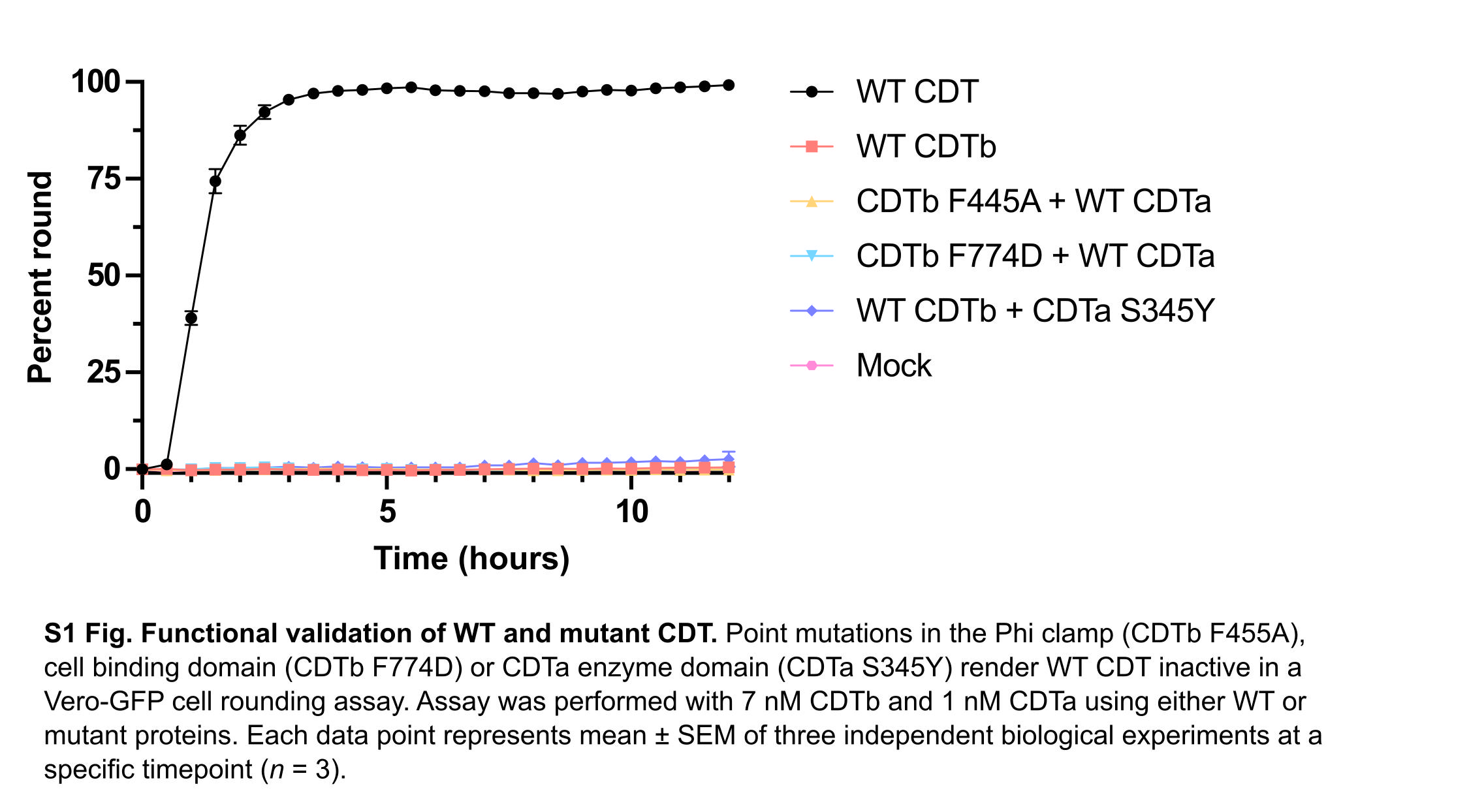

Supplement: S1 Fig — Point mutations in the Phi clamp (CDTb F455A), cell binding domain (CDTb F774D) or CDTa enzyme domain (CDTa S345Y) render WT CDT inactive in a Vero-GFP cell rounding assay. Assay was performed with 7 nM CDTb and 1 nM CDTa using either WT or mutant proteins. Each data point represents mean ± SEM of three independent biological experiments at a specific timepoint (n = 3). (TIF) [file ppat.1012568.s001.tif]

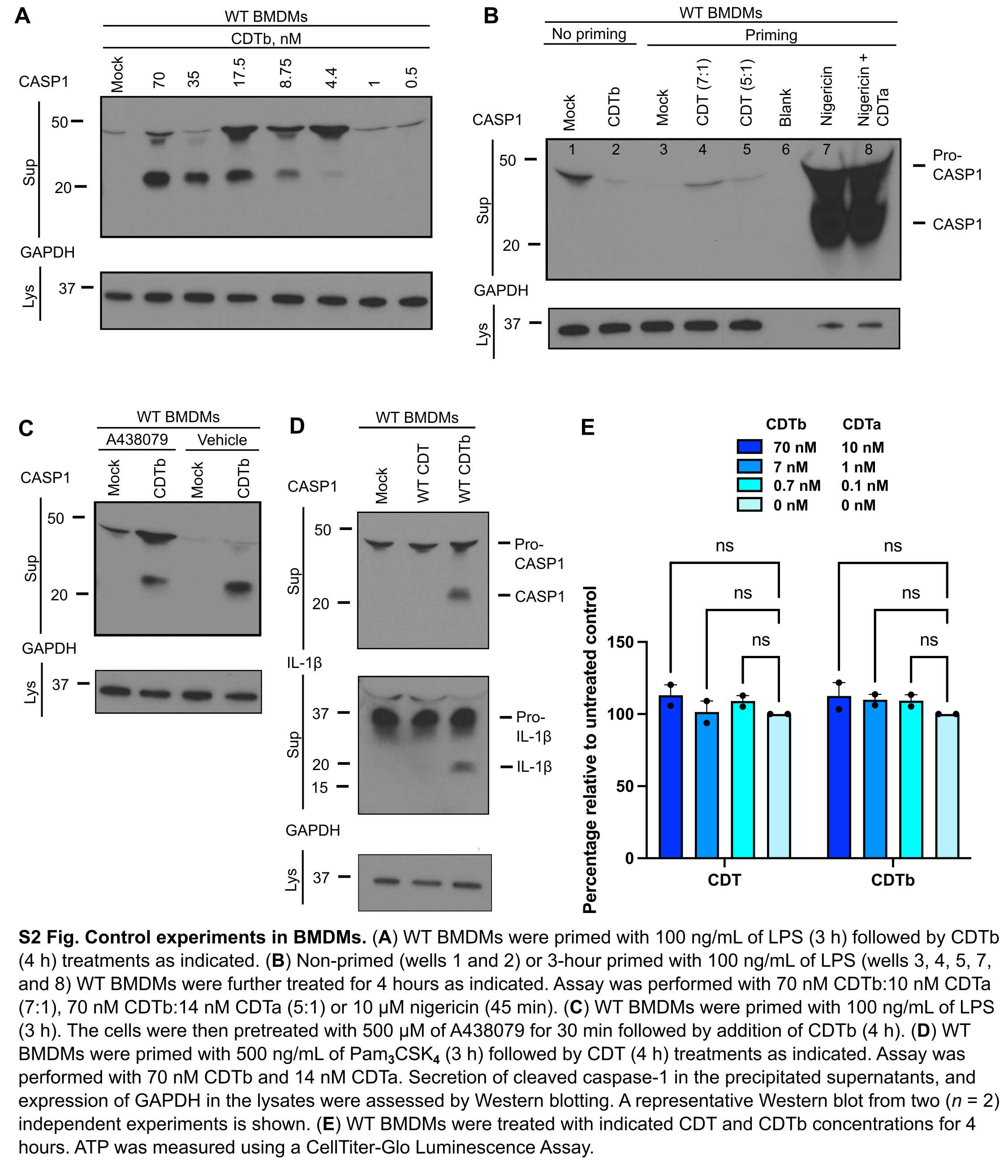

Supplement: S2 Fig — (A) WT BMDMs were primed with 100 ng/mL of LPS (3 h) followed by CDTb (4 h) treatments as indicated. (B) Non-primed (wells 1 and 2) or 3-hour primed with 100 ng/mL of LPS (wells 3, 4, 5, 7, and 8) WT BMDMs were further treated for 4 hours as indicated. Assay was performed with 70 nM CDTb:10 nM CDTa (7:1), 70 nM CDTb:14 nM CDTa (5:1) or 10 μM nigericin (45 min). (C) WT BMDMs were primed with 100 ng/mL of LPS (3 h). The cells were then pretreated with 500 μM of A438079 for 30 min followed by addition of CDTb (4 h). (D) WT BMDMs were primed with 500 ng/mL of Pam3CSK4 (3 h) followed by CDT (4 h) treatments as indicated. Assay was performed with 70 nM CDTb and 14 nM CDTa. Secretion of cleaved caspase-1 in the precipitated supernatants, and expression of GAPDH in the lysates were assessed by Western blotting. A representative Western blot from two (n = 2) independent experiments is shown. (E) WT BMDMs were treated with indicated CDT and CDTb concentrations for 4 hours. ATP was measured using a CellTiter-Glo Luminescence Assay. (TIF) [file ppat.1012568.s002.tif]

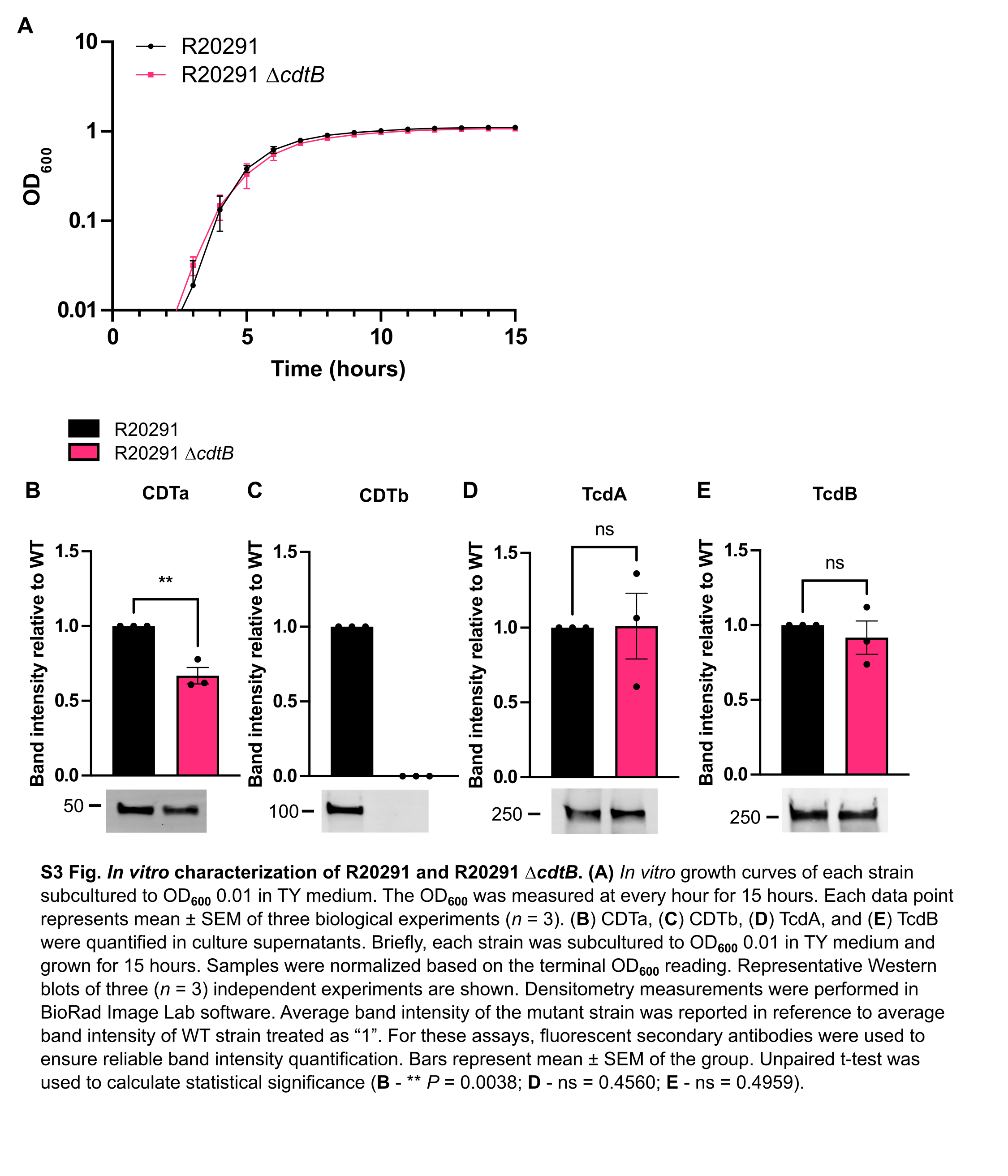

Supplement: S3 Fig — (A) In vitro growth curves of each strain subcultured to OD600 0.01 in TY medium. The OD600 was measured at every hour for 15 hours. Each data point represents mean ± SEM of three biological experiments (n = 3). (B) CDTa, (C) CDTb, (D) TcdA, and (E) TcdB were quantified in culture supernatants. Briefly, each strain was subcultured to OD600 0.01 in TY medium and grown for 22 hours. Samples were normalized based on the terminal OD600 reading. Representative Western blots of three (n = 3) independent experiments are shown. Densitometry measurements were performed in BioRad Image Lab software. Average band intensity of the mutant strain was reported in reference to average band intensity of WT strain treated as “1”. For these assays, fluorescent secondary antibodies were used to ensure reliable band intensity quantification. Bars represent mean ± SEM of the group. Unpaired t-test was used to calculate statistical significance (B—** P = 0.0038; D—ns = 0.4560; E—ns = 0.4959). (TIF) [file ppat.1012568.s003.tif]

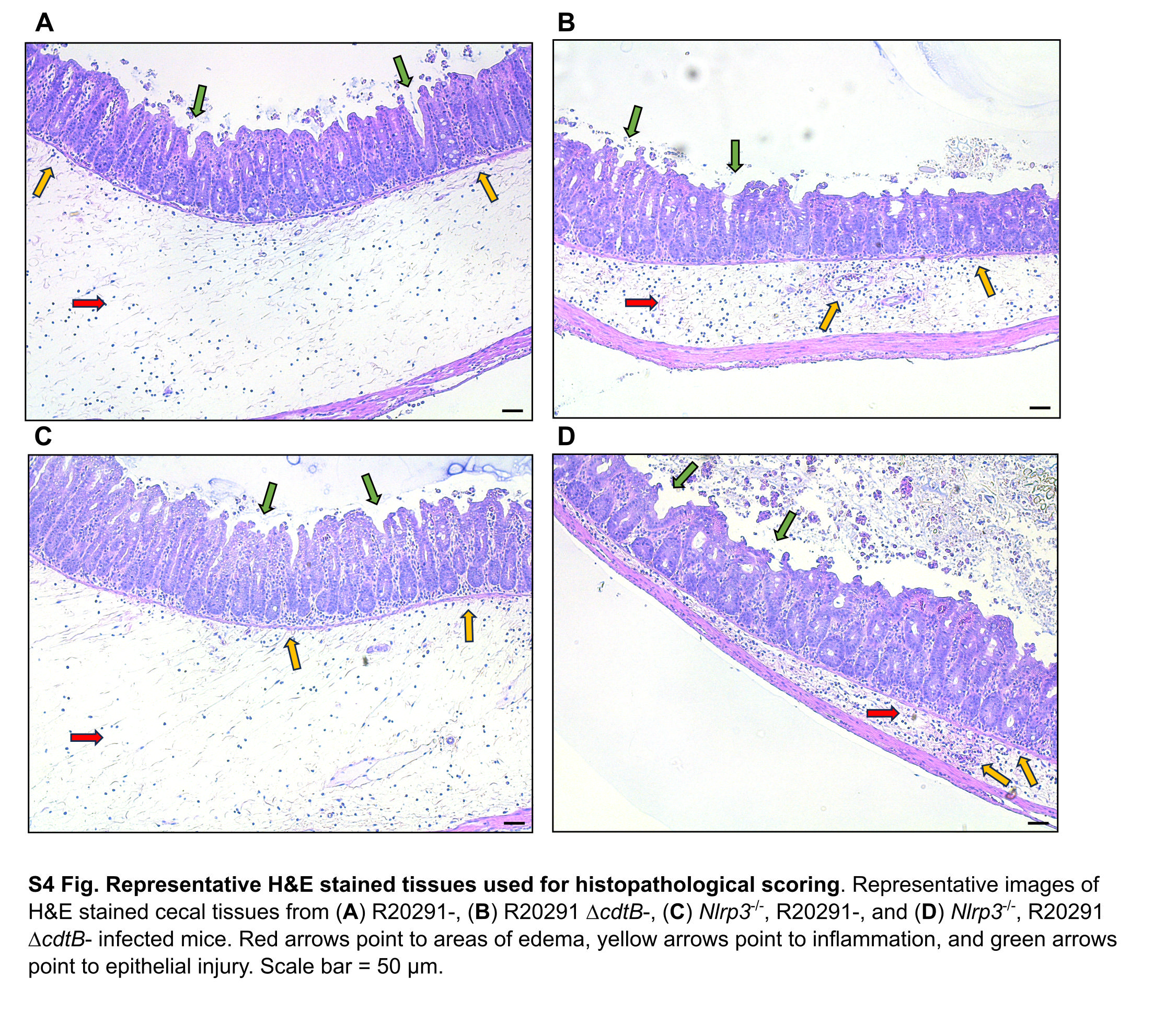

Supplement: S4 Fig — Representative images of H&E stained cecal tissues from (A) R20291-, (B) R20291 ΔcdtB-, (C) Nlrp3-/-, R20291-, and (D) Nlrp3-/-, R20291 ΔcdtB- infected mice. Red arrows point to areas of edema, yellow arrows point to inflammation, and green arrows point to epithelial injury. Scale bar = 50 μm. (TIF) [file ppat.1012568.s004.tif]

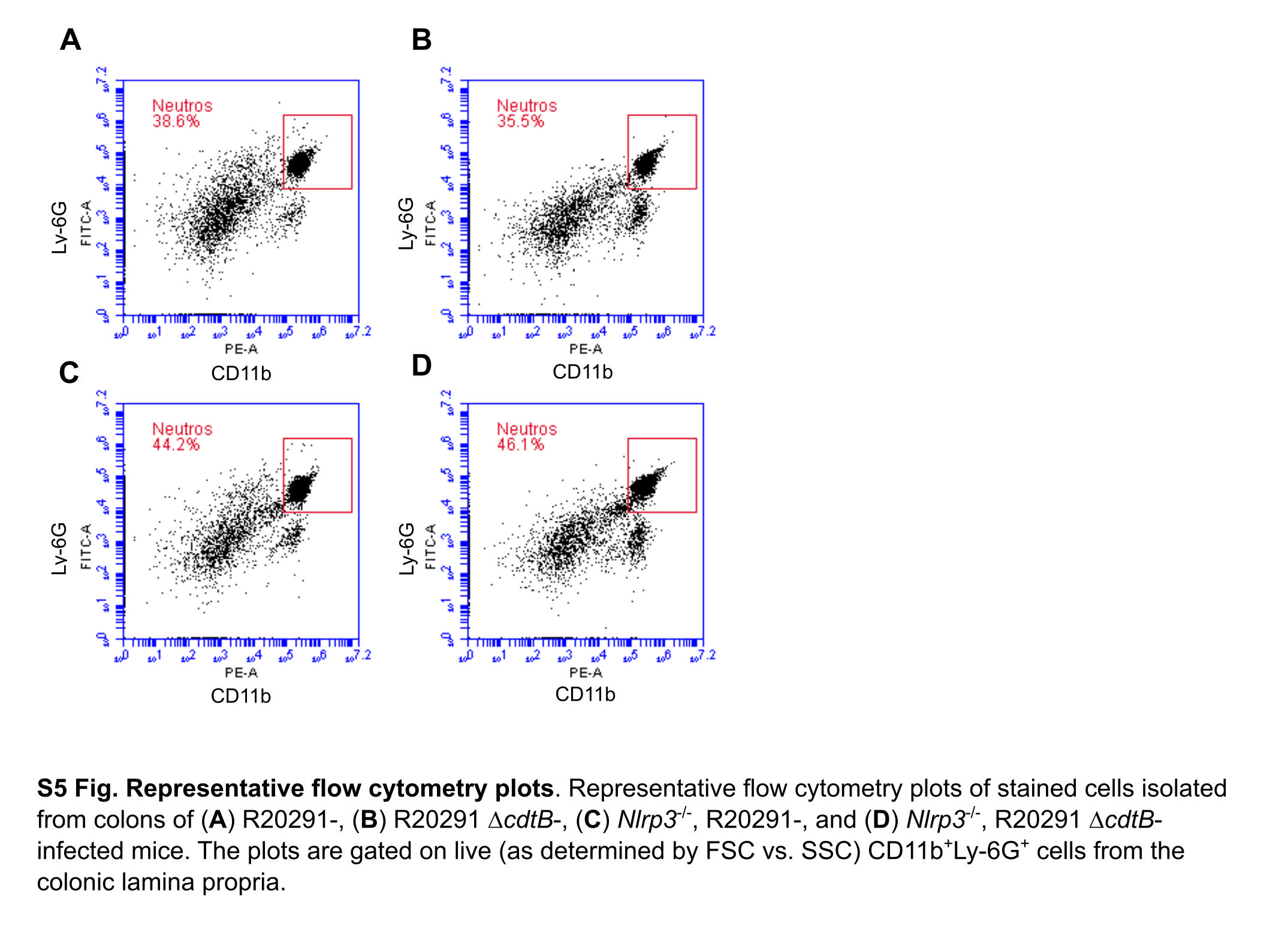

Supplement: S5 Fig — Representative flow cytometry plots of stained cells isolated from colons of (A) R20291-, (B) R20291 ΔcdtB-, (C) Nlrp3-/-, R20291-, and (D) Nlrp3-/-, R20291 ΔcdtB- infected mice. The plots are gated on live (as determined by FSC vs. SSC) CD11b+Ly-6G+ cells from the colonic lamina propria. (TIF) [file ppat.1012568.s005.tif]

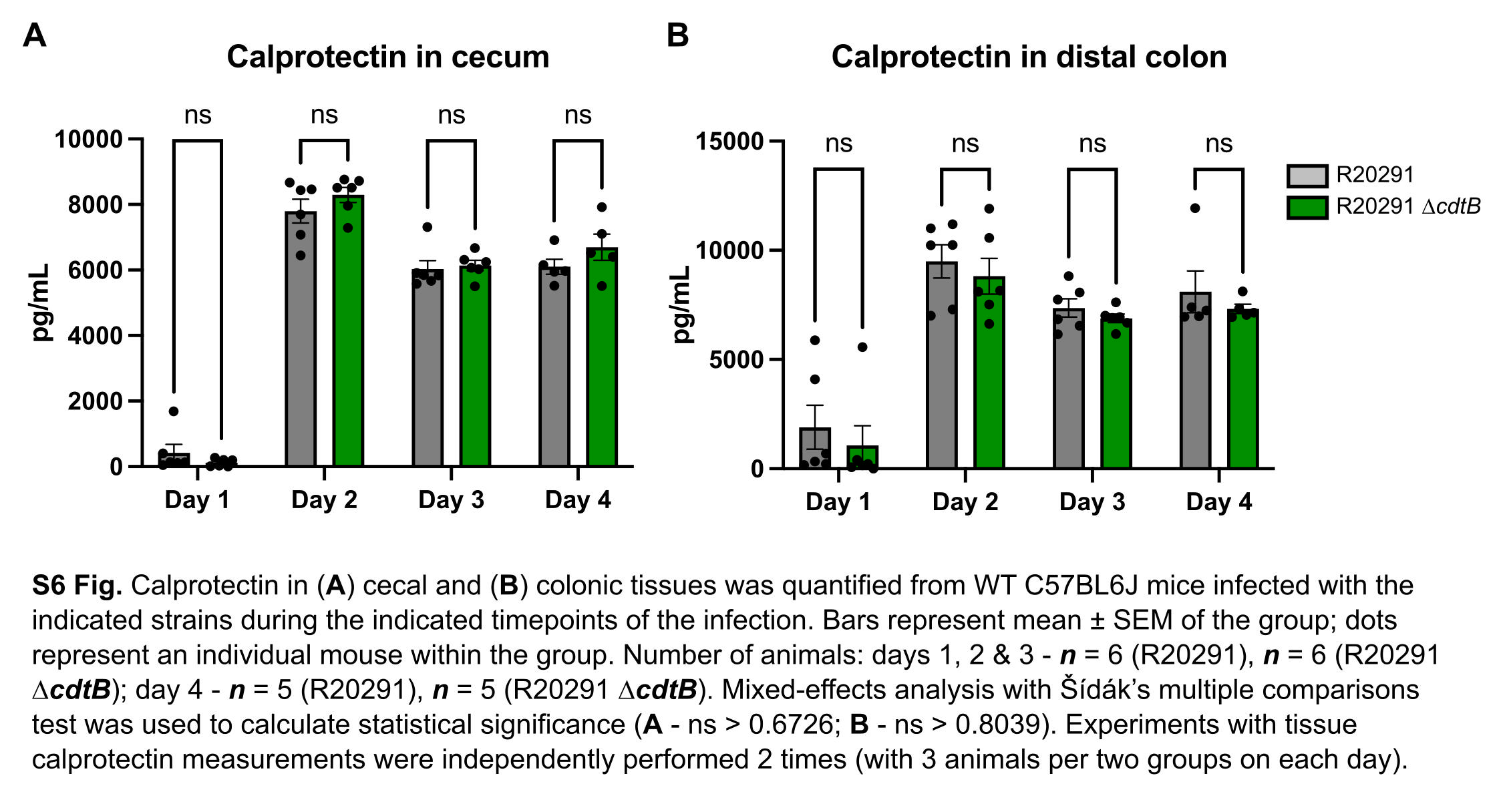

Supplement: S6 Fig — Calprotectin in (A) cecal and (B) colonic tissues was quantified from WT C57BL6J mice infected with the indicated strains during the indicated timepoints of the infection. Bars represent mean ± SEM of the group; dots represent an individual mouse within the group. Number of animals: days 1, 2 & 3—n = 6 (R20291), n = 6 (R20291 ΔcdtB); day 4—n = 5 (R20291), n = 5 (R20291 ΔcdtB). Mixed-effects analysis with Šídák’s multiple comparisons test was used to calculate statistical significance (A—ns > 0.6726; B—ns > 0.8039). Experiments with tissue calprotectin measurements were independently performed 2 times (with 3 animals per two groups on each day). (TIF) [file ppat.1012568.s006.tif]

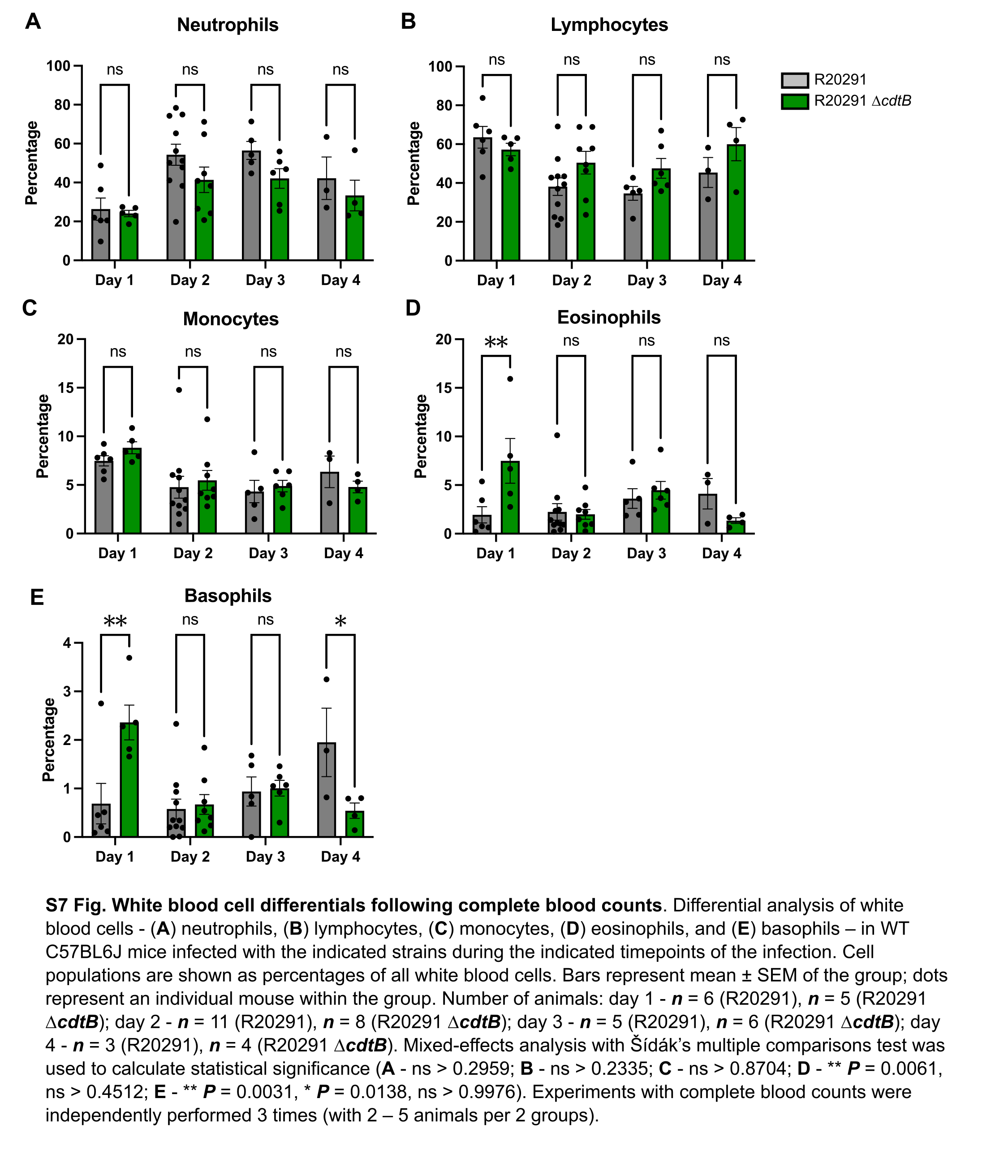

Supplement: S7 Fig — Differential analysis of white blood cells—(A) neutrophils, (B) lymphocytes, (C) monocytes, (D) eosinophils, and (E) basophils–in WT C57BL6J mice infected with the indicated strains during the indicated timepoints of the infection. Cell populations are shown as percentages of all white blood cells. Bars represent mean ± SEM of the group; dots represent an individual mouse within the group. Number of animals: day 1—n = 6 (R20291), n = 5 (R20291 ΔcdtB); day 2—n = 11 (R20291), n = 8 (R20291 ΔcdtB); day 3—n = 5 (R20291), n = 6 (R20291 ΔcdtB); day 4—n = 3 (R20291), n = 4 (R20291 ΔcdtB). Mixed-effects analysis with Šídák’s multiple comparisons test was used to calculate statistical significance (A—ns > 0.2959; B—ns > 0.2335; C—ns > 0.8704; D—** P = 0.0061, ns > 0.4512; E—** P = 0.0031, * P = 0.0138, ns > 0.9976). Experiments with complete blood counts were independently performed 3 times (with 2–5 animals per 2 groups). (TIF) [file ppat.1012568.s007.tif]
